# Physics-guided residual learning for phase-aware UAV trajectory prediction in urban environments

**DOI:** 10.1038/s41598-026-67222-5

**Published:** 2026-08-28

**Authors:** Md Ashraful Islam, Stanley Förster, Tianxiong Zhang, Hartmut Fricke

**Affiliations:** 1 Chair of Air Transport Technology and Logistics, Technical University of Dresden, Dresden, Germany; 2 Chair of Software Technology, Technical University of Dresden, Dresden, Germany

**Keywords:** UAV, Trajectory prediction, Safety, Residual learning, PIML, Engineering, Mathematics and computing

## Abstract

Reliable and accurate trajectory prediction is critical for the safe and efficient operation of unmanned aerial vehicles (UAVs) in complex urban environments, where flight dynamics are subject to wind disturbances, dense obstacle fields, and strongly phase-dependent behavior. Conventional physics-based approaches are limited by parameter uncertainty, simplifying assumptions, and unmodeled disturbances, while data-driven models may lack physical plausibility and robustness across different flight phases. To address these limitations, this paper proposes a physics-guided hybrid residual-correction framework for UAV trajectory prediction. The approach combines a physics-based model with a data-driven sequence model and learns a residual correction that compensates for the deviation between analytical prediction and observed flight behavior. In addition, the model is trained with physics-guided feasibility regularization to promote realistic speed, acceleration, jerk, and landing descent behavior. Experimental evaluation on a real-world test set shows that the proposed method yields improved results relative to the stand-alone physics model, the LSTM model, and an MLP-based fusion model across all major metrics, including RMSE, MAE, ADE, and FDE. Phase-wise analysis further demonstrates strong improvements in cruise and landing, while highlighting takeoff as the most challenging phase. The results indicate that combining physical structure with learned residual correction provides a more accurate, physically consistent, and operationally interpretable approach for UAV trajectory forecasting.

## Introduction

Unmanned Aerial Vehicles (UAVs) are becoming an integral component of emerging urban operations, including surveillance, emergency response, delivery, and the future vision of Urban Air Mobility (UAM)^1,2^. In these applications, accurate trajectory prediction is a foundational requirement for safe and efficient navigation, conflict detection, collision risk assessment, flight planning, airspace management, and safety assurance and autonomous decision-making^3,4^. Unlike open airspace, urban environments present unique challenges due to the presence of buildings, constrained flight corridors, turbulent and spatially varying wind fields, and rapidly changing operational conditions, especially during takeoff and landing^5^. Therefore, trajectory prediction models developed under simplified assumptions may not achieve the level of accuracy, robustness, and physical consistency required for safety-critical UAV operations in complex urban environments.

The main objective of this study is to develop and evaluate a phase-aware UAV trajectory prediction framework for urban environments to improve trajectory forecasting not only in terms of overall prediction accuracy but also in terms of reliability during the most dynamic and safety-critical phases of flight, such as takeoff and landing. This study explicitly considers the heterogeneous nature of urban UAV operations by evaluating how different modeling approaches capture phase-specific flight behavior under class imbalance, wind disturbances, and physical feasibility requirements, with the aim of predicting an accurate, robust, and operationally meaningful trajectory for safety-critical UAV deployment.

From a modeling perspective, UAV trajectory prediction has traditionally been approached from two largely separate directions. On the one hand, physics-based methods rely on kinematic or dynamic equations to propagate motion in a mechanistically interpretable way, often using smoothness-aware trajectory generation and control formulations^6^. These methods preserve consistency with known vehicle dynamics and are attractive for analysis and control integration. However, their predictive performance can degrade in practice because real flight behavior is shaped not only by nominal rigid-body dynamics, but also by closed-loop control actions, uncertain aerodynamic parameters, wind disturbances, and site-specific environmental effects that are difficult to model explicitly^7^. On the other hand, data-driven methods can learn nonlinear temporal dependencies directly from telemetry and have shown promise in aircraft and UAV trajectory forecasting^8^. Nevertheless, purely data-driven predictors may require substantial representative data, may generalize poorly outside training conditions, and may produce trajectories that are statistically accurate but physically implausible. A key challenge in UAV operations is observed in the significant variation of UAV motion across different flight phases, such as takeoff, cruise, and landing^9^. The dynamics during takeoff, cruise, and landing are distinct and should not be treated as a single homogeneous regime^10,11^. Takeoff is characterized by rapid vertical ascent and control transients; cruise is generally smoother and more stable; landing involves descent, deceleration, and heightened sensitivity to wind, positioning errors, and obstacle proximity. These differences are particularly consequential in urban settings, where prediction errors during takeoff and landing can directly impact operational safety due to proximity to ground infrastructure, vertiports, and obstacles^12,13^. This highlights a key methodological gap between predictive flexibility and physical realism. Another important challenge in real-world UAV trajectory prediction is class imbalance. In typical flight datasets, the cruise phase represents most of the recorded data, while takeoff and landing phases are less frequent but more dynamic and safety-critical^14,15^. As a result, models trained on imbalanced data may achieve good overall accuracy but perform poorly during these minority phases. Addressing this issue is therefore important not only from a machine learning perspective, but also for reliable and safe UAV operation^16,17^. To address these gaps, Physics-Informed Machine Learning (PIML) provides a useful direction by combining data-driven learning with physical constraints. PIML has recently emerged as a promising alternative, integrating physical structure into data-driven learning to retain the adaptability of neural models while constraining predictions to remain consistent with UAV motion principles^18,19^. By embedding UAV motion principles into the learning process, PIML can improve robustness under limited or imbalanced data, reduce physically inconsistent predictions, and support better generalization across flight phases and environmental conditions^20,21^. Therefore, comparing a deterministic physics-based model, a purely data-driven, and an MLP provides a structured basis for evaluating the trade-offs between interpretability, prediction accuracy, robustness, and physical feasibility.

This study addresses this problem by proposing a physics-informed hybrid trajectory forecasting framework that combines the strengths of physics-based models and data-driven learning, includes awareness of different flight phases, and considers physical feasibility to improve the accuracy, robustness, and practical reliability of UAV trajectory prediction. The main contributions of this paper are the following:

- A wind-aware physics based model is first developed to predict UAV trajectories using motion dynamics, wind-relative drag, and phase-aware assumptions.
- A data-driven trajectory prediction model is then developed to learn nonlinear UAV motion patterns directly from flight data, capturing behaviors that may not be fully represented by the analytical physics model.
- A Multi-Layer Perceptron (MLP) based fusion model is introduced by combining the outputs and features of the physics-based and data-driven models. The MLP learns the residual difference between two models, thereby improving prediction accuracy across different flight phases.
- Finally, a Physics-Guided Residual Learning (PGRL) framework is developed by embedding physical feasibility constraints into the learning objective, including speed bounds, acceleration and jerk smoothness, and landing vertical-rate constraints, to produce more realistic and stable UAV trajectory forecasts.

The subsequent sections of this article are structured in the following manner:  “Literature review” section presents the related work, reviewing existing studies on UAV trajectory prediction, physics-informed modeling, and safety-critical urban flight operations.  “Methodology” section describes the proposed methodology, including the model architecture, data processing procedure, and trajectory prediction formulation. “Experimental Framework and Assumptions” section introduces the experimental framework, including the dataset, flight-phase definition, evaluation metrics, and experimental assumptions. “Results and Discussion” section presents the results and discussion, including prediction performance, comparison with models, and physical feasibility analysis. Finally, “Conclusion” section concludes the paper by summarizing the main findings, discussing limitations, and outlining future research directions.

## Literature review

UAV trajectory prediction is essential for autonomous navigation, conflict detection, collision-risk assessment, and mission planning in dense urban airspace. Therefore, research on trajectory prediction is crucial, especially for UAVs that may operate in complex, densely populated environments. Advancements in physical and data-driven approaches have led to the development of methods like traditional physical models, classical machine learning, deep learning, and reinforcement learning models^22^ (Table 1).

**Table 1:** Classification of UAV trajectory prediction methods.

Methods	Models	Studies
Physics-based models	Kinematic, dynamic, aerodynamics, graph theory	^6,7,12,23–33^
Model predictive control (MPC)	Linear MPC, nonlinear MPC, drag-aware MPC, hybrid aerodynamics-based MPC	^34–39^
Optimization-based control	Metaheuristic algorithms, genetic algorithm, particle swarm optimization, optimal control	^8,40–43^
Traditional machine learning	Linear regression, Gaussian processes, support vector machines, Markov models	^22,44–50^
Deep learning	Recurrent neural networks, convolutional neural networks, LSTM, CNN-LSTM, GANs, Transformer	^51–64^
Reinforcement learning	Deep Q-network, policy-based methods, model-based reinforcement learning, multi-task reinforcement learning	^65–70^

Early work in UAV motion prediction and planning was dominated by physics-based formulations grounded in rigid-body dynamics, differential flatness, and optimal control^25^. Physics-based models have long served as the foundation for UAV trajectory prediction due to their interpretability and computational efficiency. Classical approaches typically employ analytical kinematic or dynamic models, often incorporating wind-aware dynamics and aerodynamic drag to simulate UAV motion^26^. Kinematic models use simplified equations of motion, while dynamic models incorporate forces, moments, and aerodynamic effects^27^. Cano Lopes et al.^68^ developed a comprehensive 6-DOF nonlinear aerodynamic model for fixed-wing UAVs, integrating wind estimation and drag modeling to achieve accurate predictions across various maneuvers. Similarly,^33^ introduced flight estimation algorithms for drag parameters and air data, improving aerodynamic efficiency without the need for wind tunnel calibration. The underlying physical models so far rely on physical principles of kinematics and kinetics^7^. Simulation platforms like ROS Gazebo have been used to test and validate these models under controlled conditions^22^. However, their accuracy diminishes in dynamic and uncertain environments due to simplified assumptions. Physics-based state estimators (e.g. Kalman filters and motion models) can predict short-term UAV motion by extrapolating dynamics, but they struggle when environmental uncertainties (wind, sudden maneuvers) violate model assumptions^22^. Extending Kalman filters can improve trajectory estimation by incorporating real-time sensor data. Despite these strengths, physics-based models are constrained by parameter deviation, simplifying assumptions (such as rigid body dynamics and steady wind), and their inability to capture unmodeled aerodynamic disturbances limitations that are particularly pronounced in complex urban environments^28^. For instance,^7^ further demonstrated that drag and wind-relative motion materially affect quadrotor behavior, indicating that simplified kinematic propagation can become inaccurate when external disturbances are strong or spatially heterogeneous.

To overcome these limitations, data-driven approaches have increasingly been used to learn trajectory evolution directly from flight histories. Machine learning-based data-driven models have gained attention for their ability to predict complex and nonlinear trajectory patterns. Ma and Tian^56^ developed a 4-D trajectory prediction model using historical flight data and UAV equations of motion, using a genetic algorithm for dynamic weighting. Wang et al.^71^ used the Gaussian process regression (GPR) algorithm to learn the state transition model for trajectory prediction. Recurrent neural networks, particularly Long Short-Term Memory (LSTM) architectures, are well suited to this task because they capture temporal dependence and nonlinear motion patterns from multivariate time-series telemetry^51^. In aerospace applications, LSTM-based predictors have demonstrated improved accuracy over simpler baselines for flight trajectory forecasting, including recent UAV-specific studies using lightweight or bidirectional LSTM designs^54,55^. Ma and Tian^72^ developed a UAV trajectory prediction model using a long short-term memory network (LSTM) to improve accuracy for up to 3 seconds look ahead time. LSTM models can capture temporal dependencies and hence predict UAV trajectories in dynamic environments, even with partial or noisy data^73^. Besides LSTMs, other machine learning techniques, like Random Forests, Gradient Boosting, Convolutional Neural Networks (CNN), and Gaussian techniques, are used for trajectory prediction^22^. However, they require large amounts of high-quality data for training and are computationally intensive, making real-time applications challenging. Hybrid CNN-LSTM models^56^ further enhance performance by extracting spatial and temporal features, while Bayesian LSTM approaches introduce uncertainty quantification for robust prediction. However, these data-driven approaches often lack physical plausibility, are prone to overfitting, and generalize poorly to unfamiliar flight scenarios, particularly under data imbalance^55^. These weaknesses are especially problematic for safety-critical urban UAV applications, where operational credibility matters as much as statistical fit. Combining models, hybrid frameworks lead to improved accuracy, robustness, and easier interpretability in complex scenarios^56^ but face integration and scalability issues^22^. Within data-driven forecasting pipelines, multilayer perceptrons (MLPs) remain important because they provide lightweight nonlinear function approximation and effective feature fusion. MLP-based fusion and correction models are increasingly used to bridge the gap between physics-based and data-driven predictions, but without explicit physical constraints, they may produce unrealistic trajectories^22,66^. In UAV trajectory prediction, MLPs are useful not mainly for learning the full time sequence by themselves, but for combining different types of input in a simple and efficient way. This makes MLPs well suited for hybrid models, where the aim is not to predict the whole motion from zero, but to improve existing model outputs with low computational effort.

Model Predictive Control (MPC) is a widely used optimal control strategy for UAV trajectory prediction and tracking, leveraging a rolling finite horizon optimization approach^35^. At each control step, MPC predicts the UAV’s future states over a finite horizon using a mathematical model of the UAV’s dynamics, then solves an optimization problem to minimize a cost function typically penalizing trajectory tracking error and control effort while enforcing system constraints such as actuator limits and collision avoidance^34^. Only the first control input from the optimized sequence is applied, and the process repeats at the next time step, allowing MPC to adapt in real time to disturbances and model inaccuracies^36^. In UAV applications, MPC enables precise trajectory planning and robust tracking by incorporating nonlinear or linearized UAV models and integrating real-time sensor feedback, as demonstrated in drag-aware and hybrid aerodynamics based MPC frameworks^37^. Panjavarnam et al.^34^ integrated a drag-aware physics model into a model predictive control (MPC) framework, enabling robust trajectory tracking and precise landings even under wind and external disturbances. However, MPC faces several limitations. The computational burden of solving optimization problems at each step can challenge real-time onboard implementation, especially for nonlinear models or long horizons^38^. MPC’s effectiveness is sensitive to model inaccuracies and unmodeled disturbances such as wind or turbulence, which can degrade performance^39^. Handling strongly nonlinear UAV dynamics often requires model simplification or nonlinear MPC, further increasing computational demands^34^.

Recent developments in autonomous systems increasingly demonstrate the importance of the prediction to planning interface, in which physically plausible trajectory predictions are directly incorporated into downstream motion planning, conflict detection, risk assessment to support safe and adaptive decision-making in dynamic environments. Lin et al.^74^ proposed a contingency-aware spatiotemporal optimization framework that integrates dynamic risk assessment with safety-corridor construction and model predictive control while considering uncertainty in the future behaviour of surrounding vehicles. In related work, Lin et al. ^75^ introduced a two-stage optimization framework in which a dynamic risk field provides a unified representation of static and dynamic obstacles, spatially feasible trajectory candidates are first generated, and their temporal profiles are subsequently optimized using space–time graphs and sequential quadratic programming. Although developed for autonomous vehicles, these methods demonstrate a transferable principle for UAV operations: interaction-aware predictions and their uncertainty envelopes can be converted into time-varying occupied volumes or risk constraints for conflict detection, collision-probability assessment, contingency generation, and trajectory replanning. This connection further motivates the use of hybrid prediction models that preserve UAV dynamics while retaining the adaptability of data-driven learning.

A persistent challenge in UAV trajectory prediction is data imbalance, particularly between flight phases. Models trained on imbalanced datasets tend to overfit to majority phases (typically cruise), resulting in poor accuracy for underrepresented but safety-critical phases like takeoff and landing^76^. This imbalance degrades both classification and regression performance, leading to unreliable predictions in critical scenarios. Tang et al.^77^ highlighted that takeoff and landing data are often underrepresented compared to cruise, leading to degraded accuracy in safety-critical phases. Xing et al.^78^ demonstrated that MLPs are sensitive to underrepresented phases and recommended oversampling and synthetic data generation to improve reliability. Zhong et al.^72^ addressed class imbalance in 4D trajectory prediction using spatio-temporal clustering and phase-specific regularization, while Crnovrsanin et al.^79^ applied SMOTE and oversampling to enhance minority-phase classification. This imbalance is particularly important because UAV motion is inherently phase dependent. Cruise typically exhibits smoother and more stable dynamics, whereas takeoff and landing involve stronger vertical motion, acceleration and deceleration effects, controller transients, and often greater sensitivity to wind and environmental disturbances. When these minority phases are underrepresented during training, the model may have fewer opportunities to learn their specific motion patterns^77^. Therefore, although the overall prediction accuracy across the full dataset may appear satisfactory, this aggregate performance can be misleading, as it may largely reflect the model’s ability to predict the dominant cruise phase rather than its robustness across all operational phases^80^. From a learning perspective, class imbalance can lead to several undesirable effects. First, the model may overfit the dominant cruise regime, producing predictions that are accurate in steady-flight segments but less reliable in the dynamically changing takeoff and landing phases. Second, the loss function may be implicitly dominated by majority phase samples, causing the model to prioritize minimization of cruise-related errors at the expense of minority phase fidelity. This creates the risk of overestimating the true operational capability of the model.

To address the limitations of both physics-based and data-driven methods, hybrid PIML frameworks have emerged as a promising solution. These approaches integrate physical structure with learned corrections, leveraging the strengths of both paradigms. The broader PINN literature showed that embedding physical laws into learning objectives can improve data efficiency, regularize solutions, and reduce unphysical predictions when observations are sparse or noisy^81^. In trajectory prediction, this idea is especially appealing because future motion is governed by both data-driven behavioral patterns and known kinematic or dynamic structure. Recent work in flight systems has begun to exploit this principle through physics-informed or hybrid predictors that integrate dynamics constraints, residual correction, or uncertainty-aware physical priors^82,83^. For UAV forecasting, such a strategy is valuable under limited and imbalanced data, since urban flight datasets are often dominated by cruise samples while takeoff and landing remain relatively scarce but safety-critical. Imbalanced time-series learning is known to bias optimization toward majority patterns, which can mask degraded minority-regime performance if evaluation relies only on global averages^84^. Gu et al.^85^ embedded conservation laws and aerodynamic effects into a physics-informed neural network (PINN) for quadrotor dynamics. Perrusquia et al.^86^ utilized control physics-informed machine learning for drone intention inference, demonstrating improved interpretability and operational reliability. Inbaraj and Chen^82^ applied PINNs for uncertainty quantification in urban air mobility, while Alam et al.^87^ proposed PINNs with discretized physics-based loss for stable, accurate trajectory prediction for vessels. Collectively, these studies show that hybrid frameworks outperform standalone models, especially in safety-critical and data-limited scenarios.

Despite substantial progress in UAV trajectory prediction, several important gaps remain in the development of robust, physically meaningful, and operationally reliable models for urban airspace. First, many existing studies rely on simulated, simplified assumptions or generated datasets, which do not fully capture real-world such as wind disturbances and phase-wise motion variability. Second, most trajectory prediction frameworks treat UAV motion as a homogeneous sequence and do not explicitly account for the phase-dependent behavior of takeoff, cruise, and landing. As a result, prediction performance in safety-critical phases is often insufficiently evaluated, even though these phases involve stronger vertical motion, acceleration changes, and higher operational risk. Third, purely physics-based models offer interpretability but often depend on simplified kinematic or dynamic assumptions, while purely data-driven models can capture nonlinear motion patterns but may generate physically unrealistic trajectories. Finally, existing physics-informed machine learning frameworks also remain limited in jointly addressing phase-awareness, class imbalance, and physical feasibility constraints. To address these limitations, this study develops a phase-aware physics-informed hybrid trajectory prediction framework trained and evaluated using real-world UAV data. The proposed approach combines physics-based prediction, data-driven learning that learns context-dependent corrections from physics outputs, data-driven predictions, phase information, and operating conditions. By improving prediction accuracy, robustness, and physical plausibility, the framework also supports more reliable collision risk assessment and real-time safety for UAV operations in complex urban environments.

## Methodology

This study proposes a robust hybrid trajectory prediction framework for UAVs operating in complex urban environments. The framework combines a physics-based UAV dynamics model with a data-driven Long Short-Term Memory (LSTM) network, while a neural Multi-Layer Perceptron (MLP) is employed to fuse the outputs of both components and generate the final trajectory prediction. In this way, the proposed architecture exploits both the interpretability and physical consistency of first-principles flight dynamics and the adaptive pattern-learning capability of historical flight data^56,88^. Furthermore, a Physics-Guided Residual Learning (PGRL) model is developed to enhance UAV trajectory prediction, enabling the framework to better represent realistic UAV motion in challenging urban scenarios.

### Physics-based trajectory model

#### Dynamic model formulation

To accurately represent quadcopter motion, this study develops a six-degree-of-freedom (6-DOF) gray-box dynamic model. The model captures both the translational motion of the UAV in three-dimensional space and its rotational motion about the body axes. Thus, it describes not only the vehicle’s movement along the *x*, *y*, and *z* directions, but also its change in orientation throughout flight^24,29,30,89^. The model operates in closed loop: a phase-aware reference trajectory is first constructed from the observed motion, and a nonlinear thrust-and-attitude controller then tracks this reference while the full rigid-body dynamics are numerically integrated. In this way, the predicted position, velocity, attitude, and angular velocity always evolve according to the physical equations of motion rather than being read directly from the kinematic trend.

The UAV state is defined by the following state vector:

1 $$\begin{aligned} X = \begin{bmatrix} p \\ v \\ q \\ \omega \end{bmatrix} \in {\mathbb {R}}^{13} \end{aligned}$$

where $p = [x, y, z]^T$ represents the UAV position in the inertial frame, $v = [v_x, v_y, v_z]^T$ denotes the linear velocity in the inertial frame, $q = [q_w, q_x, q_y, q_z]^T$ is the unit quaternion representing the UAV attitude, and $\omega = [\omega _x, \omega _y, \omega _z]^T$ denotes the angular velocity in the body frame. A local Cartesian East–North–Up (ENU) inertial frame is used, with the positive *z*-axis directed upward, and the quaternion rotates vectors from the body frame to the inertial frame. The state-space formulation provides a complete description of UAV motion while avoiding the singularity issues commonly associated with Euler-angle representations^24,89^. Specifically, the position and velocity states characterize the translational behavior of the vehicle, whereas the quaternion and angular velocity states describe its rotational dynamics and attitude evolution.

#### Aerodynamic drag and wind

Aerodynamic drag represents the resistive force generated by the interaction between the UAV and the surrounding air during flight. This force acts in the direction opposite to the relative motion of the vehicle with respect to the wind and therefore reduces the UAV’s translational speed. Because the projected area and aerodynamic response of a quadcopter differ between the horizontal and vertical directions, the drag is modeled anisotropically. Denoting the relative airspeed by $v_{r} = v - v_{\mathrm{wind}}$, the horizontal and vertical drag components are formulated as:

2 $$\begin{aligned} & {\mathbf{F}}_{\mathrm{drag},xy} = -\frac{1}{2}\rho C_{D,xy} A_{xy} \left\| v_{r,xy} \right\| \begin{bmatrix} v_{r,x}&v_{r,y}&0 \end{bmatrix}^{T}, \end{aligned}$$

3 $$\begin{aligned} & {\mathbf{F}}_{\mathrm{drag},z} = -\frac{1}{2}\rho C_{D,z} A_{z} \left| v_{r,z} \right| \begin{bmatrix} 0&0&v_{r,z} \end{bmatrix}^{T}, \end{aligned}$$

4 $$\begin{aligned} & {\mathbf{F}}_{\mathrm{drag}} = {\mathbf{F}}_{\mathrm{drag},xy} + {\mathbf{F}}_{\mathrm{drag},z}, \end{aligned}$$

where *ρ * is the air density, $C_{D,xy}$ and $C_{D,z}$ are dimensionless drag coefficients, and $A_{xy}$ and $A_{z}$ are the corresponding reference areas. The term $v - v_{\mathrm{wind}}$ corresponds to the relative airspeed, that is, the UAV velocity measured with respect to the moving air rather than the ground. The drag force increases quadratically with the magnitude of the relative airspeed^7^ and always acts opposite to the direction of motion through the air. As a result, stronger wind disturbances or higher UAV speeds lead to larger drag forces. Including this term allows the dynamic model to better capture realistic flight behavior, particularly in urban environments where wind effects can significantly influence trajectory evolution^27^.

The wind acting on the vehicle is derived from the available meteorological measurements. The measured wind speed $V_w$ and direction $\chi _w$ are converted into an inertial-frame mean wind vector,

5 $$\begin{aligned} v_{\mathrm{wind}} = k_w V_w \begin{bmatrix} \cos \chi _w&\sin \chi _w&0 \end{bmatrix}^{T}, \quad k_w = 0.25, \end{aligned}$$

where the attenuation factor $k_w$ scales the station-level wind measurement to an effective near-ground value experienced by the UAV. The wind vector is held constant within each prediction segment, and no unobserved random turbulence is injected during evaluation^32,90,91^.

#### Equations of motion

The UAV motion is governed by a set of nonlinear equations that describe the evolution of position, velocity, angular velocity, and orientation over time. These equations capture both the translational and rotational dynamics of the quadcopter and form the basis of the physics-based trajectory model^24,29,30^. The translational motion is given by

6 $$\begin{aligned} {\dot{p}} = v \end{aligned}$$

which states that the rate of change of position is equal to the UAV velocity. The velocity dynamics are expressed as

7 $$\begin{aligned} {\dot{v}} = \frac{1}{m}\left( T R(q) e_3 + {\mathbf{F}}_{\mathrm{drag}} \right) - g e_3 \end{aligned}$$

where *g* denotes gravitational acceleration, *m* is the UAV mass, *R*(*q*) is the rotation matrix corresponding to the quaternion *q*, $e_3 = [0,0,1]^T$, and *T* is the collective thrust magnitude acting along the positive body *z*-axis, bounded by $0 \le T \le T_{\max }$. This equation indicates that the UAV acceleration is determined by the combined effects of gravity, thrust, and drag, and the explicit frame convention removes any ambiguity in the signs of the gravity and thrust terms^30^. The rotational dynamics are described by Euler’s rigid-body equation,

8 $$\begin{aligned} {\dot{\omega }} = I^{-1}\left( \tau - \omega \times (I\omega ) \right) \end{aligned}$$

where $I = \operatorname {diag}(I_{xx}, I_{yy}, I_{zz})$ is the body-frame inertia matrix, *τ * is the control torque, and *ω * is the angular velocity vector. This equation governs how the UAV rotates in response to applied torques while accounting for gyroscopic coupling effects. Finally, the attitude dynamics are represented using quaternion propagation:

9 $$\begin{aligned} {\dot{q}} = \frac{1}{2} q \otimes \begin{bmatrix} 0 \\ \omega \end{bmatrix} \end{aligned}$$

where *⊗ * denotes quaternion multiplication. This formulation updates the UAV orientation over time based on its angular velocity, and the multiplication order is consistent with a body-to-inertial quaternion and an angular velocity expressed in the body frame. The quaternion representation is adopted because it provides a smooth and singularity-free description of rotational motion^89^.

#### Phase-aware reference trajectory

Since each flight phase has different operational characteristics, the prediction model is designed in a phase-dependent manner so that it can adapt its behavior according to the current flight condition, representing the natural progression of UAV motion from takeoff to cruise and landing^26,92^. The phase label of each segment is supplied by the segmented data and is not inferred or optimized during prediction.

For each takeoff, cruise, or landing segment, the initial velocity ${\hat{v}}_0$ and acceleration ${\hat{a}}_0$ are estimated by fitting a second-order polynomial independently to each position coordinate over the initial observation window. Direct open-loop integration of such noisy measured accelerations produces substantial drift over long horizons. To reduce this drift, the estimated acceleration is first clipped component-wise to a physically plausible interval of $\pm 0.40\,\mathrm {m\,s^{-2}}$ and then exponentially damped during propagation:

10 $$\begin{aligned} a_{r}(t) = \gamma _s {\hat{a}}_0 \exp \left( -\frac{t}{\tau _a}\right) , \quad s \in \{\text {takeoff}, \text {cruise}, \text {landing}\}, \end{aligned}$$

where $\gamma _s$ is a phase-dependent acceleration scale. This treatment preserves the short-term motion information contained in the observed acceleration while progressively reducing its influence at longer horizons. Analytical integration gives the reference velocity,

11 $$\begin{aligned} v_{r}(t) = {\hat{v}}_0 + \gamma _s {\hat{a}}_0 \tau _a \left( 1 - \exp \left[ -\frac{t}{\tau _a}\right] \right) , \end{aligned}$$

and the reference position,

12 $$\begin{aligned} p_{r}(t) = p_0 + {\hat{v}}_0 t + \gamma _s {\hat{a}}_0 \tau _a \left[ t - \tau _a \left( 1 - \exp \left[ -\frac{t}{\tau _a}\right] \right) \right] . \end{aligned}$$

where ${\mathbf{v}}_{r}(t)$ and ${\mathbf{p}}_{r}(t)$ denote the reference velocity and reference position at prediction time *t*, respectively. The vector ${\mathbf{p}}_{0}$ is the initial UAV position, while $\hat{{\mathbf{v}}}_{0}$ and $\hat{{\mathbf{a}}}_{0}$ are the estimated initial velocity and acceleration obtained from the available state measurements. The parameter $\gamma _{s}$ is a phase-dependent acceleration scaling coefficient, with the subscript *s* indicating the corresponding flight phase, whereas $\tau _{a}$ is the acceleration-decay time constant. Phase-specific vertical constraints are subsequently imposed to encode the characteristic motion of each phase. During takeoff, the initial vertical velocity is constrained to the positive interval; during landing, it is constrained to the bounded negative interval; and during cruise, near-zero vertical motion is enforced.

#### Tracking control

A nonlinear tracking controller converts the reference trajectory into thrust and torque commands. The commanded inertial acceleration combines the feedforward reference acceleration with proportional–derivative feedback on the position and velocity tracking errors,

13 $$\begin{aligned} a_{c} = a_{r} + K_p \left( p_{r} - p \right) + K_v \left( v_{r} - v \right) , \end{aligned}$$

where $K_p$ and $K_v$ are the position and velocity gain matrices, with larger vertical gains reflecting the tighter altitude regulation of multirotor flight. Each component of $a_c$ is limited to specific value to avoid commanding overly aggressive maneuvers and to promote smooth flight^26^. The desired total force then compensates gravity and aerodynamic drag,

14 $$\begin{aligned} F_{d} = m\left( a_{c} + g e_3 \right) - {\mathbf{F}}_{\mathrm{drag}}, \end{aligned}$$

from which the bounded collective thrust is obtained as

15 $$\begin{aligned} T = \operatorname {clip}\left( \left\| F_{d} \right\| _2,\, 0,\, T_{\max } \right) . \end{aligned}$$

where *T* denotes the commanded collective thrust magnitude. The vector ${\mathbf{F}}_{d}$ represents the desired total force generated by the controller. The desired body *z*-axis is aligned with $F_d$ and is tilted back toward the vertical whenever the commanded tilt exceeds the maximum tilt angle of the platform. Combining this direction with the yaw angle extracted from the initial attitude of the segment, which is held as the yaw reference throughout the forecast to maintain stable heading behavior, yields the desired attitude $q_d$. The attitude error is defined geometrically on *SO*(3) using the rotation-vector logarithm,

16 $$\begin{aligned} e_R = \operatorname {Log}\left( R(q)^T R(q_d) \right) , \end{aligned}$$

and the control torque consists of proportional attitude feedback, angular-rate damping, and a passive rotational damping term:

17 $$\begin{aligned} \tau = I \left( K_R e_R - K_\omega \omega \right) - c_\omega \omega , \end{aligned}$$

where $K_R$ and $K_\omega$ are the attitude and rate gains and $c_\omega$ is the rotational damping coefficient. During landing, the phase-specific vertical reference constraints give vertical motion and attitude stability higher importance, while during cruise the damped acceleration prior promotes smoother motion and lower control effort.

#### Numerical integration

The complete closed-loop model is propagated with the fourth-order Runge–Kutta (RK4) integration method, which provides a good balance between numerical accuracy and computational efficiency, making it suitable for predicting UAV motion over short time steps^93,94^. In general, the UAV state evolves according to a system of ordinary differential equations (ODEs), which can be written as

18 $$\begin{aligned} \frac{dy}{dt} = f(t, y) \end{aligned}$$

where *y* represents the system state. For the UAV model, the function *f*(*t*, *y*) computes the time derivatives of the state variables as

19 $$\begin{aligned} f(t,y) = \begin{bmatrix} {\dot{p}} \\ {\dot{v}} \\ {\dot{q}} \\ {\dot{\omega }} \end{bmatrix} \end{aligned}$$

where ${\dot{p}}$, ${\dot{v}}$, ${\dot{q}}$, and ${\dot{\omega }}$ denote the rates of change of position, velocity, orientation, and angular velocity obtained from the closed-loop equations of motion, with the thrust and torque commands recomputed at every function evaluation. Given the current time *t*, the current state *y*(*t*), and the integration time step *h*, the RK4 method computes four intermediate slope estimates:

20 $$\begin{aligned} & k_1 = f(t, y) \end{aligned}$$

21 $$\begin{aligned} & k_2 = f\left( t + \frac{h}{2},\, y + \frac{h}{2} k_1\right) \end{aligned}$$

22 $$\begin{aligned} & k_3 = f\left( t + \frac{h}{2},\, y + \frac{h}{2} k_2\right) \end{aligned}$$

23 $$\begin{aligned} & k_4 = f\left( t + h,\, y + h k_3\right) \end{aligned}$$

These four terms represent derivative evaluations at different intermediate points within the same time step. The next state is then obtained by combining them as

24 $$\begin{aligned} y(t+h) = y(t) + \frac{h}{6}\left( k_1 + 2k_2 + 2k_3 + k_4 \right) \end{aligned}$$

The RK4 integrator therefore evaluates the UAV dynamics several times within a single step, rather than only once at the beginning of the step, which improves the accuracy of state propagation, especially for the nonlinear dynamics of quadcopter motion^31^. After each update, the quaternion subvector is renormalized to preserve the unit-norm constraint.

### Data-driven LSTM model

#### Data preprocessing input representation

The dataset comprises multivariate time-series from UAV flights, where each record includes a set of *p=14* physical and operational features such as kinematic states, wind conditions, battery metrics, and payload parameters^57,95^. Each sample is further annotated with categorical phase label indicating the operational stage (*takeoff*, *cruise*, or *landing*). To ensure data reliability, we discard samples with excessive missing values:

25 $$\begin{aligned} \text {If } \mathrm{NaN}_\text {row} > 0.1p, \quad \text {then row is removed} \end{aligned}$$

where $\mathrm{NaN}_\text {row}$ counts missing features in that row. Remaining missing entries are imputed via forward then backward filling to preserve temporal continuity:

26 $$\begin{aligned} X_{t,i} = {\left\{ \begin{array}{ll} X_{t-1,i}, & \text {if } X_{t,i} \text { is missing} \\ X_{t+1,i}, & \text {if still missing} \end{array}\right. } \end{aligned}$$

To reduce the impact of outliers, each feature is clipped between its empirical 1st and 99th percentiles:

27 $$\begin{aligned} X_{t,i} = \min \!\bigl (\max (X_{t,i}, Q_{0.01,i}), Q_{0.99,i}\bigr ) \end{aligned}$$

where $Q_{q,i}$ denotes the *q*-quantile of feature *i*^96^. To include flight-phase information in the model, the categorical phase variable is represented using encoding^97^ where each sample belongs to one of three flight phases: takeoff, cruise, or landing. The encoded phase vector is written as

28 $$\begin{aligned} P_t = \left[ p^{\mathrm{takeoff}}_t,\; p^{\mathrm{cruise}}_t,\; p^{\mathrm{landing}}_t\right] \end{aligned}$$

where, for example, $p^{\mathrm{takeoff}}_t = 1$ if sample *t* belongs to the takeoff phase, and 0 otherwise. In the same way, the cruise and landing indicators identify the corresponding flight phase. The final input feature vector is formed by combining the numerical flight-state variables with the encoded phase information:

29 $$\begin{aligned} X_t = \left[ x_{t,1}, x_{t,2}, \ldots , x_{t,14}, p^{\mathrm{takeoff}}_t, p^{\mathrm{cruise}}_t, p^{\mathrm{landing}}_t\right] \in {\mathbb {R}}^{17} \end{aligned}$$

#### Group-based data split and sequence construction

To avoid overlap between the training and testing subsets, the dataset is split on the basis of entire flight missions rather than individual samples^80^. Let *F* denote the set of all unique flights. A group-based split is then defined as

30 $$\begin{aligned} & F_{\mathrm{train}} = \{\text {first 280 flights}\} \end{aligned}$$

31 $$\begin{aligned} & F_{\mathrm{test}} = F \setminus F_{\mathrm{train}} \end{aligned}$$

As a result, each flight is assigned completely either to the training set or to the test set. This ensures that samples from the same flight do not appear in both subsets, which would otherwise lead to overly optimistic prediction performance. After the train–test split, min–max normalization is applied using statistics computed only from the training set:

32 $$\begin{aligned} X'_{t,i} = \frac{ X_{t,i} - \min _{\tau \in T_{\mathrm{train}}} X_{\tau ,i} }{ \max _{\tau \in T_{\mathrm{train}}} X_{\tau ,i} - \min _{\tau \in T_{\mathrm{train}}} X_{\tau ,i} } \end{aligned}$$

where $T_{\mathrm{train}}$ denotes the set of training time indices. This normalization scales each feature to a comparable range and improves the stability of the training process^98^. To capture temporal dependencies in UAV motion, a sliding-window strategy is used to construct input sequences^14^. For each time step *t*, the model receives the previous *L* input vectors as a sequence:

33 $$\begin{aligned} S_t = [X_{t-L}, \ldots , X_{t-1}] \in {\mathbb {R}}^{L \times 17} \end{aligned}$$

This sequence serves as the model input and summarizes the recent flight history over the prediction horizon. Based on this sequence, the model predicts the UAV position at the next time step:

34 $$\begin{aligned} y_t = \left[ \mathrm{position}_x,\; \mathrm{position}_y,\; \mathrm{position}_z\right] _t \in {\mathbb {R}}^3 \end{aligned}$$

#### LSTM-based model architecture

The trajectory prediction model is built using a Long Short-Term Memory (LSTM) network^99^, followed by a dropout layer^100^, a dense hidden layer with ReLU activation^101^, and an output layer. This architecture is designed to capture temporal dependencies in UAV flight sequences while reducing overfitting and improving prediction performance. Given an input sequence $S_t$, the LSTM first extracts a latent temporal representation:

35 $$\begin{aligned} h_t = \mathrm{LSTM}_{\theta }(S_t) \end{aligned}$$

where *θ * denotes the trainable parameters of the LSTM model. The hidden representation $h_t$ summarizes the relevant motion patterns contained in the input sequence. To improve generalization and reduce overfitting, dropout is then applied to the LSTM output:

36 $$\begin{aligned} h'_t = \mathrm{Dropout}(h_t, p) \end{aligned}$$

where *p* is the dropout rate. This step randomly deactivates a fraction of the hidden units during training, encouraging the model to learn more robust features. The resulting feature vector is passed through a dense layer with a rectified linear unit (ReLU) activation:

37 $$\begin{aligned} z_t = \mathrm{ReLU}(W_d h'_t + b_d) \end{aligned}$$

where $W_d$ and $b_d$ are the weight matrix and bias vector of the dense layer. This layer provides an additional nonlinear transformation of the learned temporal features. Finally, the predicted output is obtained through a linear output layer:

38 $$\begin{aligned} {\hat{y}}_t = W_o z_t + b_o \end{aligned}$$

where $W_o$ and $b_o$ denote the output-layer parameters. The final output ${\hat{y}}_t$ corresponds to the predicted UAV position at the next time step^95^.

#### Hyperparameter optimization

To identify an effective model configuration, Bayesian hyperparameter optimization is performed using Optuna^102^. The objective is to minimize the validation loss, defined as the error distance between the predicted and true UAV positions:

39 $$\begin{aligned} L(\theta ) = \frac{1}{|T_{\mathrm{val}}|} \sum _{t \in T_{\mathrm{val}}} \left\| y_t - {\hat{y}}_t \right\| _2 \end{aligned}$$

where $T_{\mathrm{val}}$ denotes the set of validation samples. The optimization procedure searches over several key hyperparameters, including the sequence length, the number of LSTM units, the number of dense-layer units, and the dropout rate. The search ranges are defined as

40 $$\begin{aligned} & L \in \{10, 20, 30, 40, 60\} \end{aligned}$$

41 $$\begin{aligned} & U_{\mathrm{lstm}} \in \{32, 64, 128\} \end{aligned}$$

42 $$\begin{aligned} & U_{\mathrm{dense}} \in \{16, 32, 64\} \end{aligned}$$

43 $$\begin{aligned} & p \sim {\mathcal {U}}(0.1, 0.5) \end{aligned}$$

where *L* is the sequence length, $U_{\mathrm{lstm}}$ is the number of hidden units in the LSTM layer, $U_{\mathrm{dense}}$ is the number of units in the dense layer, and *p* is the dropout rate sampled from a uniform distribution^102^. The Optuna search was intentionally limited to a moderate range to control training cost. The selected LSTM hidden size of was located at the upper boundary of the tested range, indicating that larger network sizes may provide additional capacity.

### Hybrid fusion via adaptive MLP

#### Parallel hybrid fusion concept

At each prediction time step, two separate estimates of the UAV next position are available. The first estimate, denoted by $x_{\mathrm{phy}} \in {\mathbb {R}}^3$, is generated by the physics-based model, while the second estimate, denoted by $x_{\mathrm{lstm}} \in {\mathbb {R}}^3$, is produced by the data-driven LSTM model. Thus, the hybrid framework has access to both a physically grounded prediction and a data-adaptive prediction at the same time. A simple way to combine these two outputs would be to use a fixed weighted average^66,103^:

44 $$\begin{aligned} {\hat{x}}_{\mathrm{next}} = w_{\mathrm{phy}} x_{\mathrm{phy}} + w_{\mathrm{lstm}} x_{\mathrm{lstm}} \end{aligned}$$

subject to

45 $$\begin{aligned} w_{\mathrm{phy}} + w_{\mathrm{lstm}} = 1 \end{aligned}$$

where $w_{\mathrm{phy}}$ and $w_{\mathrm{lstm}}$ are constant fusion weights. However, such a static combination is limited because it assumes that the contribution of each model should remain the same under all operating conditions. In practice, the relative accuracy of the physics-based and data-driven models may vary depending on the flight phase and local operating conditions. Therefore, a fixed-weight fusion strategy is not sufficiently flexible for realistic UAV trajectory prediction.

#### Adaptive fusion via multi-layer perceptron

To overcome the limitation of fixed fusion weights, the proposed framework uses a trainable Multi-Layer Perceptron (MLP) to combine the outputs of the two prediction models. Instead of manually selecting how much importance to assign to each model, the MLP learns the fusion rule directly from data^104,105^. This adaptive fusion mechanism allows the framework to adjust the balance between the physics-based and LSTM-based predictions according to the current prediction context. For example, under some conditions the physics-based estimate may be more reliable because it preserves dynamic consistency, while under other conditions the LSTM estimate may better capture complex patterns learned from historical flight data. By learning this relationship automatically, the MLP can provide a more effective combination than a simple fixed average.

#### Input representation

The input to the fusion MLP is formed by concatenating the outputs of the two individual models:

46 $$\begin{aligned} z = \begin{bmatrix} x_{\mathrm{phy}} \\ x_{\mathrm{lstm}} \end{bmatrix} \in {\mathbb {R}}^6 \end{aligned}$$

where the first three elements correspond to the physics-based prediction and the next three elements correspond to the LSTM-based prediction. In this way, the fusion network receives both physically constrained information and data-driven trajectory estimates as input. Based on this combined representation, the MLP learns to generate the final predicted UAV position. Therefore, the proposed hybrid fusion framework integrates the strengths of both modeling approaches, namely the interpretability and physical consistency of the physics-based model and the adaptability of the data-driven LSTM model.

#### Network architecture and loss function

The fusion model is implemented as a Multi-Layer Perceptron (MLP) that takes the combined prediction vector from the physics-based and LSTM-based models as input. The input layer has dimension 6, corresponding to the concatenated outputs of the two individual predictors. This input is then processed through two fully connected hidden layers followed by a rectified linear unit (ReLU) activation function. The final output layer produces the predicted UAV position at the next time step:

47 $$\begin{aligned} {\hat{x}}_{\mathrm{next}} = f_{\mathrm{MLP}}(z) \in {\mathbb {R}}^3 \end{aligned}$$

where *z* is the fusion input vector and $f_{\mathrm{MLP}}(\cdot )$ denotes the nonlinear mapping learned by the network^106,107^. The fusion MLP is trained to predict the true next UAV position by minimizing a mean squared error (MSE) loss between the predicted output and the ground-truth position:

48 $$\begin{aligned} L = \frac{1}{M} \sum _{i=1}^{M} \left\| x^{(i)}_{\mathrm{true}} - {\hat{x}}^{(i)}_{\mathrm{next}} \right\| ^2 \end{aligned}$$

where *M* is the mini-batch size, $x^{(i)}_{\mathrm{true}}$ is the true next position for sample *i*, and ${\hat{x}}^{(i)}_{\mathrm{next}}$ is the corresponding fused prediction^98^. The loss function encourages the model to reduce the average prediction error over all samples in the batch. In this way, the training process not only improves prediction accuracy but can also promote physically meaningful trajectory outputs^50^.

### Physics-guided residual learning model

To integrate the complementary strengths of physics-based and data-driven prediction, a physics-guided residual-correction framework (Fig 5) is developed in which the data-driven LSTM prediction serves as the trajectory backbone, while the physics-based model acts as a structured guidance signal: it enters the model as an input feature and as a consistency term in the training objective, and a learned gate controls how strongly a physics-informed correction is applied to the backbone trajectory^108,109^.

#### Residual-correction concept

Instead of directly replacing either prediction, the proposed method models the final trajectory as the sum of a refined data-driven backbone and a physics-informed residual correction:

49 $$\begin{aligned} {\hat{P}}_k = f_{\mathrm{bb}}\!\left( {\hat{P}}^{\mathrm{lstm}}_k\right) + G_k \odot \Delta P_k, \quad \Delta P_k = f_{\mathrm{corr}}(Z_k), \quad G_k = \sigma \!\left( f_{\mathrm{gate}}(Z_k)\right) , \end{aligned}$$

where ${\hat{P}}^{\mathrm{lstm}}_k = \{{\hat{p}}^{\mathrm{lstm}}(k,\tau )\}_{\tau =1}^{H}$ is the LSTM-predicted position horizon, $f_{\mathrm{bb}}(\cdot )$ is a backbone network that refines this data-driven horizon, $f_{\mathrm{corr}}(\cdot )$ is a correction network, $f_{\mathrm{gate}}(\cdot )$ is a gating network, $\sigma (\cdot )$ is the element-wise sigmoid function, *⊙ * denotes the Hadamard product, and *H* is the prediction horizon^110,111^. The gate $G_k \in (0,1)^{H\times 3}$ adaptively controls, for each future step and spatial axis, how much of the learned correction is added to the backbone. In this way, the strong short-horizon accuracy of the data-driven predictor is preserved, while the neural correction focuses on compensating systematic mismatch where the two predictors disagree, making the learning task more structured and physically grounded than a purely data-driven trajectory predictor^112^.

#### Input representation and network architecture

The model receives two horizon-wise sources of information: the data-driven LSTM prediction ${\hat{P}}^{\mathrm{lstm}}_k$ and a sample-conditioned physics-based prior ${\tilde{P}}^{\mathrm{phy}}_k = \{{\tilde{p}}^{\mathrm{phy}}(k,\tau )\}_{\tau =1}^{H}$, with ${\hat{p}}^{\mathrm{lstm}}(k,\tau ), {\tilde{p}}^{\mathrm{phy}}(k,\tau ) \in {\mathbb {R}}^{3}$. The physics-based prior is obtained from the phase-dependent physics-model rollout, interpolated onto the horizon time grid and conditioned to each sample by matching its initial position to the first LSTM-predicted position:

50 $$\begin{aligned} {\tilde{p}}^{\mathrm{phy}}(k,\tau ) = p^{\mathrm{phy}}_{\pi _k}(\tau ) - p^{\mathrm{phy}}_{\pi _k}(1) + {\hat{p}}^{\mathrm{lstm}}(k,1), \end{aligned}$$

where $\pi _k$ denotes the flight phase of sample *k*. During landing, the vertical component is additionally rescaled so that the total descent of the prior matches the descent indicated by the LSTM horizon,

51 $$\begin{aligned} \begin{aligned} s_k&= \operatorname {clip}\!\left( \frac{ {\hat{z}}^{\mathrm{lstm}}(k,1) - {\hat{z}}^{\mathrm{lstm}}(k,H) }{ {\tilde{z}}^{\mathrm{phy}}(k,1) - {\tilde{z}}^{\mathrm{phy}}(k,H) }, \,0.35,\,1.65 \right) , \\ {\tilde{z}}^{\mathrm{phy}}(k,\tau )&\leftarrow {\tilde{z}}^{\mathrm{phy}}(k,1) - s_k \left[ {\tilde{z}}^{\mathrm{phy}}(k,1) - {\tilde{z}}^{\mathrm{phy}}(k,\tau ) \right] . \end{aligned} \end{aligned}$$

where *k* denotes the current prediction instance, $\tau \in \{1,\ldots ,H\}$ is the forecast-step index, and *H* is the total number of steps in the prediction horizon. The joint feature representation combines both horizons with their element-wise disagreement and sum,

52 $$\begin{aligned} Z_k = \left[ \, {\tilde{P}}^{\mathrm{phy}}_k,\; {\hat{P}}^{\mathrm{lstm}}_k,\; {\tilde{P}}^{\mathrm{phy}}_k - {\hat{P}}^{\mathrm{lstm}}_k,\; {\tilde{P}}^{\mathrm{phy}}_k + {\hat{P}}^{\mathrm{lstm}}_k \,\right] \in {\mathbb {R}}^{H \times 12}, \end{aligned}$$

where the disagreement term ${\tilde{P}}^{\mathrm{phy}}_k - {\hat{P}}^{\mathrm{lstm}}_k$ provides a horizon-wise measure of the deviation between the analytical prior and the data-driven prediction and enables the network to identify systematic model mismatch. The purpose of the correction network is not to generate the entire trajectory from scratch, but to refine the backbone by compensating for systematic mismatch. Because the correction is learned jointly with the physics-based prior, the network can capture complex effects that are difficult to represent explicitly in the physical model^28,112^.

#### Training objectives and loss

The proposed hybrid model is trained for multi-step trajectory forecasting using an objective function that combines prediction accuracy with physical feasibility constraints. The goal is to ensure that the corrected trajectory is not only close to the observed data, but also remains physically plausible. Let $p_{k+\tau }$ denote the ground-truth UAV position at future step *k+τ * and let $\gamma \in (0,1]$ be a horizon discount factor. The multi-step prediction error is defined as

53 $$\begin{aligned} L_{\mathrm{data}} = \frac{1}{N} \sum _k \sum _{\tau =1}^{H} \gamma ^{\tau -1} \left\| p_{k+\tau } - {\hat{p}}_{k+\tau |k} \right\| _2^2, \end{aligned}$$

where *N* is the number of training samples and the discounting gives slightly larger weight to near-future steps^113^. This term is evaluated in standardized coordinates, whereas all feasibility penalties below are evaluated in metric coordinates. To keep the corrected trajectory close to the physically structured prior, a physics-guidance term is defined as

54 $$\begin{aligned} L_{\mathrm{phy}} = \frac{1}{N} \sum _k \sum _{\tau =1}^{H} \gamma ^{\tau -1} \left\| {\hat{p}}_{k+\tau |k} - {\tilde{p}}^{\mathrm{phy}}(k,\tau ) \right\| _2^2. \end{aligned}$$

Let the predicted position increment be

55 $$\begin{aligned} \Delta {\hat{p}}_{k+\tau |k} = {\hat{p}}_{k+\tau |k} - {\hat{p}}_{k+\tau -1|k}. \end{aligned}$$

A speed-bound penalty^81^ is then defined as

56 $$\begin{aligned} L_{\mathrm{spd}} = \frac{1}{N} \sum _k \sum _{\tau =2}^{H} \left[ \max \left( 0, \frac{\Vert \Delta {\hat{p}}_{k+\tau |k}\Vert _2}{\Delta t} - v_{\max }(\pi _k) \right) \right] ^2 \end{aligned}$$

where $v_{\max }(\pi _k)$ is the phase-dependent maximum speed. To promote smooth motion, second- and third-order trajectory differences are also penalized. These correspond to acceleration and jerk regularization^114^:

57 $$\begin{aligned} & \Delta ^2 {\hat{p}}_{k+\tau |k} = {\hat{p}}_{k+\tau |k} - 2{\hat{p}}_{k+\tau -1|k} + {\hat{p}}_{k+\tau -2|k} \end{aligned}$$

58 $$\begin{aligned} & \Delta ^3 {\hat{p}}_{k+\tau |k} = {\hat{p}}_{k+\tau |k} - 3{\hat{p}}_{k+\tau -1|k} + 3{\hat{p}}_{k+\tau -2|k} - {\hat{p}}_{k+\tau -3|k} \end{aligned}$$

with corresponding penalties

59 $$\begin{aligned} & L_{\mathrm{acc}} = \frac{1}{N} \sum _k \sum _{\tau =3}^{H} \left\| \Delta ^2 {\hat{p}}_{k+\tau |k} \right\| _2^2 \end{aligned}$$

60 $$\begin{aligned} & L_{\mathrm{jrk}} = \frac{1}{N} \sum _k \sum _{\tau =4}^{H} \left\| \Delta ^3 {\hat{p}}_{k+\tau |k} \right\| _2^2. \end{aligned}$$

In addition, an explicit acceleration-bound penalty constrains the implied acceleration ${\hat{a}}_{k+\tau |k} = \Delta ^2 {\hat{p}}_{k+\tau |k}/\Delta t^2$ componentwise and in magnitude:

61 $$\begin{aligned} L_{\mathrm{bnd}} = \frac{1}{N} \sum _k \sum _{\tau =3}^{H} \Big [ \max \!\big (0, |{\hat{a}}_{xy}| - a_{xy,\max }\big )^2 + \max \!\big (0, |{\hat{a}}_{z}| - a_{z,\max }\big )^2 + \max \!\big (0, \Vert {\hat{a}}_{k+\tau |k}\Vert _2 - a_{\max }\big )^2 \Big ], \end{aligned}$$

where $a_{xy,\max }$, $a_{z,\max }$, and $a_{\max }$ denote the horizontal, vertical, and total acceleration limits. Finally, a vertical-rate penalty enforces phase-dependent two-sided bounds on the predicted vertical rate ${\hat{v}}_{z} = \Delta {\hat{z}}_{k+\tau |k}/\Delta t$ during the safety-critical phases:

62 $$\begin{aligned} L_{v_z} = \frac{1}{N} \sum _k \sum _{\tau =2}^{H} {\left\{ \begin{array}{ll} \max \!\big (0,\, v_{z,\min }^{\mathrm{TO}} - {\hat{v}}_z\big )^2 + \max \!\big (0,\, {\hat{v}}_z - v_{z,\max }^{\mathrm{TO}}\big )^2, & \pi _k = \text {takeoff},\\ \max \!\big (0,\, v_{z,\min }^{\mathrm{LD}} - {\hat{v}}_z\big )^2 + \max \!\big (0,\, {\hat{v}}_z - v_{z,\max }^{\mathrm{LD}}\big )^2, & \pi _k = \text {landing},\\ 0, & \pi _k = \text {cruise}, \end{array}\right. } \end{aligned}$$

where $[v_{z,\min }^{\mathrm{TO}}, v_{z,\max }^{\mathrm{TO}}]$ and $[v_{z,\min }^{\mathrm{LD}}, v_{z,\max }^{\mathrm{LD}}]$ are the allowable climb-rate and descent-rate bands during takeoff and landing, respectively. The final training objective is written as

63 $$\begin{aligned} L = L_{\mathrm{data}} + \lambda _{\mathrm{phy}} L_{\mathrm{phy}} + \lambda _{\mathrm{spd}} L_{\mathrm{spd}} + \lambda _{\mathrm{acc}} L_{\mathrm{acc}} + \lambda _{\mathrm{bnd}} L_{\mathrm{bnd}} + \lambda _{\mathrm{jrk}} L_{\mathrm{jrk}} + \lambda _{v_z} L_{v_z} \end{aligned}$$

where the coefficients $\lambda _{\mathrm{phy}}$, $\lambda _{\mathrm{spd}}$, $\lambda _{\mathrm{acc}}$, $\lambda _{\mathrm{bnd}}$, $\lambda _{\mathrm{jrk}}$, and $\lambda _{v_z}$ control the contribution of each physics-based regularization term.

### Evaluation metrics

To evaluate the predictive performance of the models, a set of error metrics that quantify translational accuracy, consistency, deviation, and robustness was employed. The use of multiple metrics provides a more comprehensive assessment of model performance across different operational objectives^115^.

#### Root mean square error (RMSE) and mean absolute error (MAE)

The final trained model is evaluated on the holdout test set using several quantitative error metrics. These metrics are used to measure how closely the predicted UAV trajectory matches the true trajectory in each spatial direction and in overall three-dimensional space^116,117^. The prediction accuracy is assessed separately for each axis using the mean absolute error (MAE) and the root mean square error (RMSE). For each spatial axis $j \in \{x, y, z\}$, the MAE is defined as

64 $$\begin{aligned} \mathrm{MAE}_j = \frac{1}{|T_{\mathrm{test}}|} \sum _{t \in T_{\mathrm{test}}} \left| y_{t,j} - {\hat{y}}_{t,j} \right| \end{aligned}$$

and the RMSE is given by

65 $$\begin{aligned} \mathrm{RMSE}_j = \sqrt{ \frac{1}{|T_{\mathrm{test}}|} \sum _{t \in T_{\mathrm{test}}} \left( y_{t,j} - {\hat{y}}_{t,j} \right) ^2 } \end{aligned}$$

where $y_{t,j}$ denotes the true position component and ${\hat{y}}_{t,j}$ denotes the predicted position component at time step *t*. These metrics show the prediction error along each coordinate direction individually. Lower values indicate better predictive accuracy^116,117^.

#### Average displacement error (ADE)

Average Displacement Error (ADE) is a standard metric in trajectory prediction and measures the mean Euclidean distance between the predicted and ground-truth positions over the full prediction horizon:

66 $$\begin{aligned} \mathrm{ADE} = \frac{1}{NT} \sum _{i=1}^{N} \sum _{t=1}^{T} \left\| \hat{{\mathbf{p}}}_{i,t} - {\mathbf{p}}_{i,t} \right\| _2, \end{aligned}$$

where $\hat{{\mathbf{p}}}_{i,t}, {\mathbf{p}}_{i,t} \in {\mathbb {R}}^{3}$ are the predicted and true 3D positions. ADE reflects the average path-following accuracy over time and is particularly useful when the entire predicted trajectory matters, such as in navigation, obstacle avoidance^115^.

#### Final displacement error (FDE)

Final Displacement Error (FDE) evaluates the Euclidean distance between the predicted and ground-truth final positions:

67 $$\begin{aligned} \mathrm{FDE} = \frac{1}{N} \sum _{i=1}^{N} \left\| \hat{{\mathbf{p}}}_{i,T} - {\mathbf{p}}_{i,T} \right\| _2. \end{aligned}$$

where $\hat{{\mathbf{p}}}_{i,t}, {\mathbf{p}}_{i,t} \in {\mathbb {R}}^{3}$ are the predicted and true 3D positions. FDE emphasizes endpoint accuracy and is particularly relevant in UAV operations where the final state is critical, such as approach stabilization, precision landing, and docking^115^.

## Experimental framework and assumptions

This section presents the experimental design adopted to evaluate the Models and analysis. It describes the dataset structure, the model configuration, and the main assumptions underlying the analysis.

### Dataset and flight phases

The dataset consists of UAV flight trajectories collected as multivariate time-series data from multiple flight missions (table 2) from secondary sources^44–46^. Each trajectory is stored as a sequence of timestamped measurements, where each row represents the UAV state at a given time instant. Each flight contains time-series measurements, including time, wind conditions, battery status, three-dimensional position, vehicle orientation, linear velocity, angular velocity, linear acceleration . The trajectory data are explicitly divided into three operational phases: takeoff, cruise, and landing. This phase labeling is important because UAV motion is not uniform throughout the mission. During takeoff, the vehicle shows strong vertical motion and increasing control activity as it departs from the ground. The cruise phase is generally characterized by smoother and more stable translational motion.

**Table 2:** Summary of the UAV trajectory dataset used in this study.

Item	Description
Dataset type	Multivariate time-series UAV flight trajectories
Number of flight	430
Avg Flight time	5min
UAVs type	DJI Matrice100
Operation type	Low-altitude urban multirotor UAV operations
Altitude range	Below 120 m AGL (typically lower during landing and takeoff)
UAV platform	Multirotor UAV
Primary disturbance	Wind-induced stochastic velocity disturbances
Environmental variables	wind_speed, wind_angle
Power-system variables	battery_voltage, battery_current
Position variables	x_position, y_position, z_position
Orientation variables	x_orientation, y_orientation, z_orientation, w
Linear velocity variables	x_twist_linear, y_twist_linear, z_twist_linear
Angular velocity variables	x_twist_angular, y_twist_angular, z_twist_angular
Linear acceleration variables	x_linear_acceleration, y_linear_acceleration, z_linear_acceleration
Observed flight phases	Takeoff, cruise, and landing
Phase-wise sample count	Takeoff: 54,144, Cruise: 153,319, Landing: 75,335

### Model configuration, parameters, and assumptions

**Table 3:** Main configuration of all models used in this study.

Model	Parameter	Value	Description
Physics-based	Prediction horizon, $T_h$	30 s	Total future prediction duration
	Time step, *Δ t*	0.1 s	Numerical-integration step
	Mass, *m*	3.6 kg	UAV mass
	Gravity, *g*	9.81 m/s^2^	Gravitational acceleration constant
	Inertia, $(I_x,I_y,I_z)$	$(0.082,\,0.082,\,0.145)$ kg m^2^	Body inertia about the principal axes
	Air density, *ρ *	1.225 kg/m^3^	Air density used in computation
	Reference drag area	$A_{xy}=0.12$ m^2^, $A_z=0.18$ m^2^	Horizontal and vertical reference areas
	Drag coefficient	$C_{d,xy}=0.90$, $C_{d,z}=1.10$	Horizontal and vertical drag coefficients
	Maximum thrust, $F_{\max }$	88.29 N	Upper bound applied to the total thrust
	Maximum tilt angle	$35^\circ$	Maximum allowable body tilt
	Angular damping	0.18 N m s	Linear damping for body angular rate
	Wind gain	0.25	Scaling factor for the wind vector
	Trend acceleration scales	TO: 0.30, CR: 0.10, LD: 0.20	Phase-dependent scaling
	Trend acceleration clip	0.40 m/s^2^	Component-wise clipping of acceleration
	Vertical speed limits	TO: 0.5 to 2.4 m/s, LD: *-2.3* to *-0.35* m/s	Bounds used to construct references
	Position-control gains	$K_{p,xy}=0.45$, $K_{p,z}=0.65$	Horizontal and vertical position gains
	Velocity-control gains	$K_{d,xy}=1.15$, $K_{d,z}=1.35$	Horizontal and vertical velocity gains
	Attitude gain	$K_R=7.0$	Quaternion-attitude feedback gain
	Angular-rate gain	$K_{\omega }=2.8$	Body-rate feedback gain
	Max reference acceleration	2.0 m/s^2^	Maximum component of the controller acceleration
LSTM	Model type	Unified encoder–decoder	One model for all flight phases
	Input window	6 s (50 steps)	Historical lookback window
	Prediction horizon	30 s (250 steps)	Future trajectory length
	Hidden size	128	Number of recurrent hidden units
	Batch size	128	Training mini-batch size
	Learning rate	$10^{-3}$	AdamW optimizer step size
	Weight decay	$10^{-6}$	AdamW weight regularization
	Gradient clipping	1.0	Maximum gradient norm
	Phase encoding	3 one-hot inputs	Represents takeoff, cruise, and landing
MLP fusion	Input vector size	3000 (12*H*)	Flattened physics, LSTM
	Network structure	3000–334–167–750	Two-hidden-layer residual fusion network
	Batch size	64	Training mini-batch size
	Learning rate	$10^{-3}$	Implemented Adam optimizer step size
	Weight decay	$10^{-5}$	Weight regularization coefficient
	Horizon discount	0.98	Weight to near-future residual errors
PGRL	Time step	0.12 s	Effective time step of the aligned horizons
	Prediction horizon	30 s (250 steps)	Total multi-step forecast duration
	Backbone input size	750 (3*H*)	Flattened 250-step LSTM-position horizon
	Joint correction input	3000 (12*H*)	Physics, LSTM, difference, and sum horizons
	Backbone network	750–128–64–750	Learns a data-backbone trajectory
	Correction network	3000–192–96–750	Learns the joint correction
	Gate network	3000–96–750	horizon-dependent correction gate
	Batch size	64	Training mini-batch size
	Learning rate	$10^{-3}$	AdamW optimizer step size
	Weight decay	$10^{-5}$	AdamW weight regularization
	Horizon discount	0.98	Gives more weight to future prediction
	Physics guidance	$\lambda _{\mathrm{phy}}=0.015$	Penalizes deviation from trajectory
	Speed penalty	$\lambda _{\mathrm{spd}}=0.03$	Penalizes phase-specific speed-limit violations
	Acceleration penalty	$\lambda _{\mathrm{acc}}=0.002$	Penalizes large second-order differences
	Acceleration-bound	$\lambda _{\mathrm{accb}}=0.002$	Penalizes acceleration-limit exceedance
	Jerk penalty	$\lambda _{\mathrm{jrk}}=0.0005$	Penalizes large third-order differences
	Vertical-rate penalty	$\lambda _{\mathrm{vz}}=0.04$	Penalizes vertical-rate violations
	Speed limits	8.0, 14.0, 5.0 m/s	Takeoff, cruise, and landing speed bounds
	Vertical-rate limits	TO: 0.5 to 2.4 m/s, LD: *-2.3* to *-0.35* m/s	Phase-specific vertical-rate bounds

The physics-based trajectory model was parameterized using a DJI Matrice 100 multirotor as the reference unmanned aerial vehicle (UAV) platform, which is a professional multirotor aircraft designed for mapping, surveillance, and advanced sensing missions. Its official specifications, including aircraft mass, maximum takeoff weight, diagonal wheelbase, maximum flight speed, maximum ascent and descent rates, wind resistance, and maximum pitch angle, provide a suitable physical basis for defining the operating envelope of the proposed physics model^118–120^. The physics-based component employed in this study is not intended to serve as a high-fidelity rotorcraft aerodynamic simulator. Rather, it is formulated as a simplified gray-box trajectory prior that captures the dominant translational dynamics relevant to short-horizon prediction, including gravitational acceleration, aerodynamic drag, thrust saturation, attitude-related tilt constraints, wind disturbances, and phase-dependent vertical-motion limits. The model does not explicitly represent rotor inflow, blade-element aerodynamics, rotor-speed-dependent forces and moments, blade-pitch dynamics, or the complete six-component aerodynamic force and moment system obtainable through Blade Element Momentum Theory (BEMT). Its primary purpose is therefore to generate a physically structured baseline trajectory that can subsequently be refined through data-driven residual correction, rather than to replace a high-fidelity rotorcraft flight dynamics or aerodynamic simulation framework. In this work, the UAV is represented as a rigid-body multirotor subject to gravity, rotor-generated thrust, aerodynamic drag, wind disturbance, and bounded control inputs. Parameters directly available from UAV documentation are used where possible, while unavailable internal parameters, such as the inertia tensor, aerodynamic drag coefficients, response time constants, and controller gains, are estimated or tuned according to standard multirotor modeling practice^24,25,33^. The robustness of the final predictions to these assumed priors is examined in Appendix A.

The prediction horizon defines the future time interval over which the physics model propagates the UAV state. A 30 s horizon is selected because it provides sufficient look ahead for short-term UAV trajectory forecasting during takeoff, cruise, and landing. This interval is long enough to capture phase transitions, wind-induced drift, and potential deviations from the nominal trajectory, while remaining computationally feasible for repeated model rollouts, using a numerical-integration time step of $\Delta t = 0.1~\mathrm{s}$. The aircraft mass is set to $m = 3.6~\mathrm{kg}$. The mass is a fundamental parameter in the translational dynamics because it determines the weight, thrust requirement, acceleration response, and vertical maneuverability of the UAV. Thus, the physics model must generate at least approximately $3.6 \times 9.81 \approx 35.3~\mathrm{N}$ of total thrust to maintain hover in the nominal no-payload configuration, well within the applied maximum thrust bound of $F_{\max } = 88.29~\mathrm{N}$. The gravitational acceleration is set to the standard near-Earth value $g = 9.81~\mathrm {m/s^2}$. The moments of inertia are set as $(I_x, I_y, I_z) = (0.082,\,0.082,\,0.145)~\mathrm {kg\,m^2}$, which describe the resistance of the UAV to angular acceleration about the roll, pitch, and yaw axes. The values $I_x = I_y = 0.082~\mathrm {kg\,m^2}$ reflect the approximate symmetry of the aircraft in the horizontal plane, while the larger yaw inertia $I_z = 0.145~\mathrm {kg\,m^2}$ accounts for the wider mass distribution around the vertical axis^24^.

The air density is considered as $\rho = 1.225~\mathrm {kg/m^3}$. This corresponds to standard sea-level atmospheric density and is used in aerodynamic drag calculations. This standard value is appropriate when local temperature, pressure, and altitude-dependent air-density variation are not explicitly modeled^121^. The aerodynamic drag is computed over reference areas of $A_{xy} = 0.12~\mathrm {m^2}$ horizontally and $A_z = 0.18~\mathrm {m^2}$ vertically, with drag coefficients $C_{d,xy} = 0.90$ and $C_{d,z} = 1.10$ that quantify the aerodynamic resistance experienced by the UAV. The vertical drag coefficient is selected to be larger than the horizontal drag coefficient, because vertical airflow interacts with the rotor disk, landing gear, and body structure differently from horizontal airflow. The maximum tilt angle is defined as $\theta _{\max } = 35^{\circ }$. Tilt angle is important because it controls the allocation of thrust between vertical lift and horizontal acceleration^119^. In this model, $35^{\circ }$ is used as a simulation upper bound to allow limited aggressive maneuvering while still preventing unrealistic body attitudes. If strict manufacturer consistency is required, this value can be reduced to $30^{\circ }$. An angular damping term of $0.18~\mathrm {N\,m\,s}$ provides linear damping on the body angular rate. The wind gain is defined as $k_w = 0.25$. This parameter scales the influence of wind disturbance on UAV motion. The use of a wind gain is motivated by the fact that the complete aerodynamic interaction between wind, rotors, UAV body, and onboard control compensation is complex. Since the UAV has an official maximum wind resistance of approximately $12~\mathrm {m/s}$^119^, the value $k_w = 0.25$ provides a conservative representation of wind-induced trajectory disturbance.

The vertical speed limits are defined as $0.5 \le v_z^{\mathrm{takeoff}} \le 2.4~\mathrm {m/s}$ and $-2.3 \le v_z^{\mathrm{landing}} \le -0.35~\mathrm {m/s}$. These bounds constrain the ascent and descent rates during takeoff and landing^118,119^. The values used in this model are intentionally conservative because takeoff and landing are safety-critical phases. Limiting vertical speed helps avoid aggressive climb or descent behavior and supports smoother trajectory prediction in urban environments. Phase-dependent trend-acceleration scales (0.30 for takeoff, 0.10 for cruise, and 0.20 for landing) are applied, with component-wise clipping at $0.40~\mathrm {m/s^2}$. The closed-loop reference tracking uses position-control gains $K_{p,xy} = 0.45$ and $K_{p,z} = 0.65$, velocity-control gains $K_{d,xy} = 1.15$ and $K_{d,z} = 1.35$, an attitude gain $K_R = 7.0$, and an angular-rate gain $K_{\omega } = 2.8$. The maximum reference acceleration produced by the controller is bounded to $2.0~\mathrm {m/s^2}$, which prevents the physics model from generating unrealistic maneuvers^118^. Although higher accelerations may be physically possible based on thrust and tilt limits, real UAV operations are constrained by flight-controller smoothing, structural safety, payload stability, and mission-level reliability. Table 3 summarizes the physics model parameters.

The LSTM trajectory prediction model was configured as a unified encoder- decoder architecture to predict UAV motion across takeoff, cruise, and landing using a unified model. A 6 s input window was used to provide look back time, including position, velocity trend, acceleration behavior, and phase-dependent movement patterns to predicts a 30 s future trajectory. The LSTM network uses 128 hidden units and two stacked recurrent layers, providing sufficient capacity to learn nonlinear temporal dependencies while avoiding excessive model complexity. A dropout rate of 0.22 was applied between layers to reduce overfitting and improve generalization to unseen flight trajectories. Training was performed with a batch size of 128 for stable and efficient optimization. The learning rate ($10^{-3}$) and weight decay ( $10^{-6}$) provided mild regularization. Gradient clipping with a threshold of 1.0 was included to prevent exploding gradients during recurrent training. Finally, flight phase information was encoded using three one-hot inputs, allowing the unified model to distinguish takeoff, cruise, and landing dynamics while still learning shared UAV motion characteristics. Table 3 summarizes the LSTM model parameters.

The fusion model is implemented using a Multi-Layer Perceptron (MLP) that combines the outputs of the physics-based model and the LSTM model over the full prediction horizon. The purpose of this fusion network is to learn a nonlinear correction mapping between the physically guided trajectory estimate and the data-driven LSTM prediction. MLP architectures were selected for the backbone, correction, and gate networks because these components do not predict the trajectory directly from raw telemetry. The MLP design also provides a relatively simple and computationally efficient correction mechanism, which reduces model complexity and the risk of overfitting given the available dataset. The input vector concatenates the physics-based and LSTM position rollouts over the horizon together with their element-wise difference features, yielding *12H = 3000* input values for *H = 250* future steps, and the output layer produces the fused three-dimensional trajectory over all 250 future steps (750 values). Two hidden layers with 334 and 167 neurons provide sufficient capacity to learn horizon-wise weighting, correction, and residual compensation between the two prediction sources, while remaining compact enough to avoid overfitting and preserve computational efficiency, giving an overall 3000–334–167–750 structure. Training uses a mini-batch size of 64 and the Adam optimizer with a learning rate of $10^{-3}$, a standard and stable choice for adaptive optimizers, together with weight decay of $\lambda = 10^{-5}$, which introduces $L_2$ regularization and discourages excessively large network weights. A horizon discount of 0.98 is applied to weight near-future residual errors more heavily during training. To construct temporally consistent training pairs, timestamps are rounded to two decimals for robust matching, and the maximum allowed alignment gap $\Delta t_{\max } = 0.08~\mathrm{s}$ defines the largest acceptable time mismatch between the physics model output, the LSTM prediction, and the corresponding ground-truth sample; samples exceeding this threshold are excluded from fusion training. Since the nominal time step is approximately $0.1~\mathrm{s}$, this tolerance is strict enough to reject poorly matched samples while still allowing minor timestamp variations. Table 3 summarizes the MLP model parameters used in this study.

The Physics-Guided Residual Learning (PGRL) model is designed as a residual fusion model that combines the complementary outputs of the physics-based predictor and the LSTM predictor. This design is motivated by the fact that the two models provide different but complementary information. The physics model imposes motion consistency through simplified UAV dynamics, while the LSTM model captures nonlinear temporal patterns learned from real flight data. To reduce optimization bias caused by the predominance of cruise samples, phase-balanced mini-batch sampling was applied during PGRL training. For each training sample belonging to phase *c*, the sampling weight was defined as $w_i = 1/N_c$, where $N_c$ is the number of training samples in that phase. Training samples were drawn using weighted random sampling with replacement, resulting in approximately equal expected contributions from takeoff, cruise, and landing during each epoch. No SMOTE or synthetic trajectory interpolation was used. Validation and test samples were evaluated using their original phase distributions. This balancing strategy is independent of the phase-aware physical-feasibility penalties, which regularize speed, acceleration, jerk, and landing descent. By combining both sources, the PGRL model can learn a correction function that reduces prediction error while maintaining physically reasonable motion behavior.

The PGRL model is organized into three sub-networks operating over the aligned 250-step horizon. A backbone network with the structure 750–128–64–750 takes the flattened 250-step LSTM position horizon (*3H = 750*) and learns a data-driven backbone trajectory. A correction network with the structure 3000–192–96–750 takes a joint input of *12H = 3000* values, formed from the physics, LSTM, difference, and sum horizons, and learns the joint residual correction. A gate network with the structure 3000–96–750 followed by a sigmoid activation produces a horizon-dependent correction gate that modulates how strongly the correction is applied at each step. The first backbone hidden layer with 128 neurons provides sufficient capacity to learn nonlinear relationships between the physics-based and LSTM-based predictions, while the second hidden layer with 64 neurons compresses this representation and encourages a compact correction mapping. A very small network may not capture the nonlinear residual relationship between the two prediction sources, whereas a very large network may overfit the training flights. Therefore, the selected architecture provides a balance between expressive power, computational efficiency, and generalization.

The PGRL model can be interpreted as a residual correction network. Instead of learning the entire trajectory independently, the model learns a correction over the baseline prediction. A general residual formulation is useful because the physics and LSTM models already provide meaningful trajectory estimates. The PGRL network therefore focuses on correcting systematic errors, reducing drift, and improving local physical consistency. Training uses a mini-batch size of 64 and the AdamW optimizer with a learning rate of $10^{-3}$ and weight decay of $10^{-5}$. The horizon discount gives slightly greater importance to near-future prediction steps than distant future steps. Since *γ = 0.98* is close to 1, all future steps remain important, but near-future states receive slightly higher weight. The PGRL loss includes physics-inspired penalty terms to encourage physically plausible trajectories. The physics-guidance term penalizes deviation from the physics-based trajectory. The speed penalty discourages predictions that exceed reasonable phase-specific velocity limits. The acceleration penalty reduces abrupt changes in velocity, the acceleration-bound term penalizes exceedance of the acceleration limit, and the jerk penalty penalizes excessive changes in acceleration. The vertical-rate penalty is particularly important during landing, where excessive descent speed can create unsafe or physically unrealistic trajectories. These penalty terms guide the model toward solutions that are both accurate and physically consistent. The phase-specific speed limits (8.0, 14.0, and $5.0~\mathrm {m/s}$ for takeoff, cruise, and landing) are used to constrain the predicted motion according to flight phase. takeoff and landing are safety-critical phases and therefore use lower speed bounds than cruise. The speed penalty is activated only when the predicted speed exceeds the corresponding phase-specific limit. As a result, the PGRL model is encouraged to remain within realistic operational speed bounds while still learning from the training data. The vertical-rate penalty, constrained by the phase-specific vertical-rate limits (0.5 to $2.4~\mathrm {m/s}$ during takeoff and *-2.3* to $-0.35~\mathrm {m/s}$ during landing), discourages the model from producing landing predictions with excessive downward velocity. It therefore improves the physical realism and operational safety of the predicted landing trajectory. Table 3 summarizes the PGRL configuration used for prediction model.

### Implementation and computational setup

All experiments were implemented in Python and executed on a Linux workstation running Ubuntu 24.04.4 LTS. The workstation was equipped with an AMD Ryzen Threadripper PRO 7965WX processor with 24 physical cores and 48 threads, 256 GB of system memory, and an NVIDIA RTX PRO 5000 Blackwell GPU with 48 GB of dedicated GPU memory. The deep learning models were trained using mini-batch optimization with GPU acceleration where available.

## Results and discussion

This section presents the experimental results of the Physics-Informed Hybrid Residual-Correction Model and discusses its predictive behavior in comparison with others approaches. The analysis combines quantitative evaluation, qualitative trajectory visualization, and comparative assessment of model variants to examine both predictive accuracy and physical realism.

### Overall quantitative performance

The overall predictive performance of the evaluated models was assessed on the test set using Root Mean Square Error (RMSE), Mean Absolute Error (MAE), Average Displacement Error (ADE), and Final Displacement Error (FDE). RMSE and MAE measure the point-wise prediction error over the forecasting horizon, while ADE measures the average trajectory deviation across the predicted sequence. FDE measures the prediction error at the final prediction step. Since this study considers UAV trajectory prediction over a 30 s horizon, these metrics together provide a clear evaluation of both average trajectory accuracy and horizon endpoint accuracy.

**Table 4:** Overall quantitative performance of the evaluated trajectory prediction models.

Model	RMSE (m)	MAE (m)	ADE (m)	FDE (m)
Physics-based	7.975	5.883	5.883	18.821
LSTM	5.611	1.841	5.522	8.430
MLP Fusion	6.058	5.260	5.260	7.998
PGRL	**4.323**	**1.395**	**4.185**	**6.370**

**Fig. 1:**
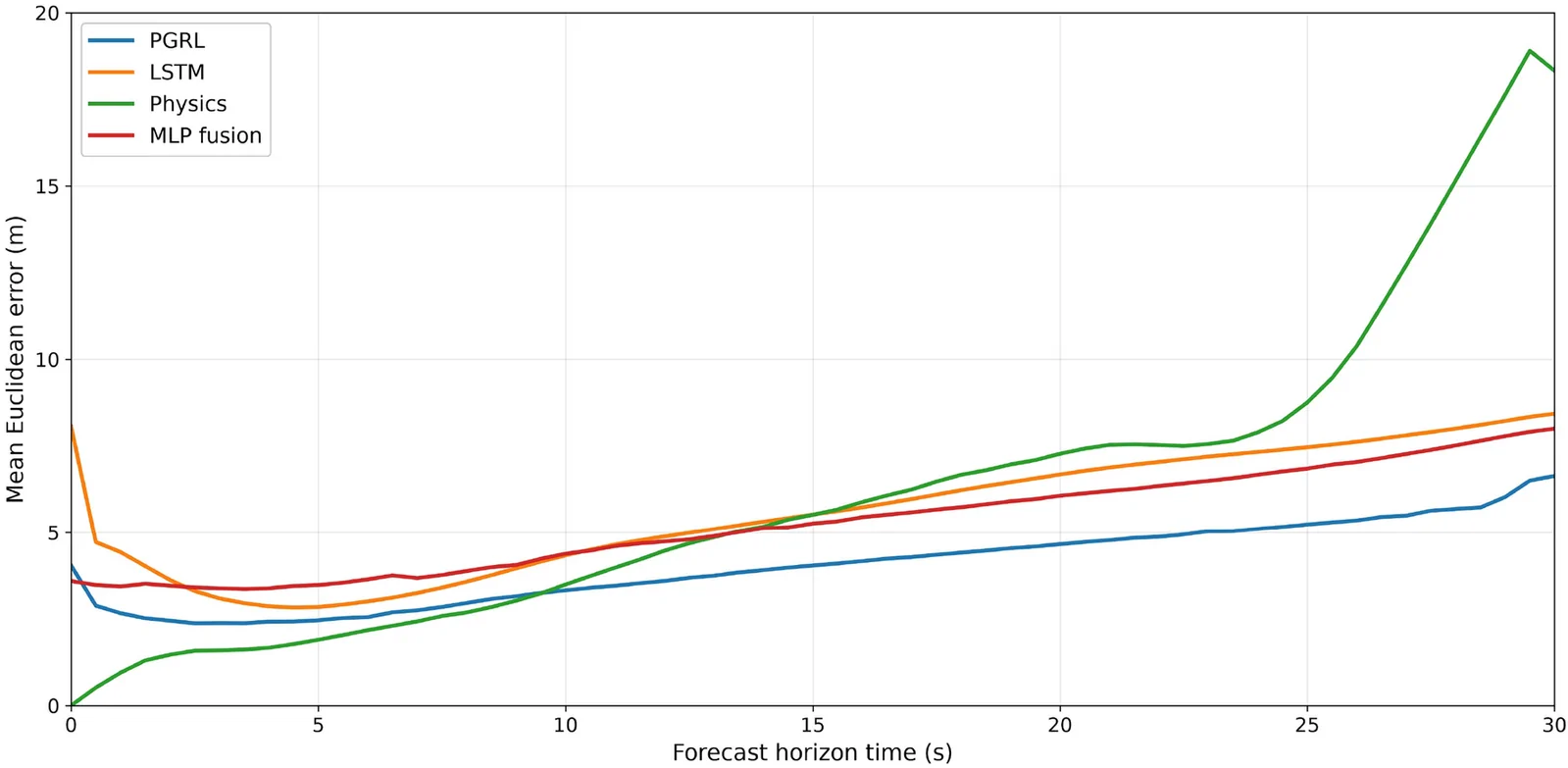
Overall error comparison of the evaluated models. The physics-based model shows the largest prediction drift, while the LSTM and PGRL models produce substantially lower errors.

Table 4 and Fig. 1 show that the standalone physics-based model has the largest prediction error among the evaluated methods. This indicates that the analytical estimation alone cannot fully capture the real UAV trajectory behavior over the 30 s prediction horizon. Although the physics-based model provides a structured motion estimate, the error increases strongly over time because the simplified dynamics cannot represent all real flight effects, such as phase transitions, control response, and disturbance-related deviations. The LSTM model substantially improves prediction accuracy compared with the physics-based model, reducing the RMSE to 5.611 m and the ADE to 5.522 m. This confirms that the data-driven model can learn dominant motion patterns from the UAV telemetry. The MLP fusion model does not improve on the LSTM overall: its RMSE (6.058 m) is higher, and while its FDE (7.998 m) is slightly lower than the LSTM’s (8.430 m), its MAE (5.260 m) are considerably worse than the corresponding LSTM values of 1.841 m. This suggests that the fusion strategy can reduce the final-horizon error, but does not consistently improve average trajectory agreement or point-wise accuracy. The proposed PGRL model achieves the best overall performance across all metrics. Compared with the physics-based model, PGRL reduces RMSE by approximately 45.8%, MAE by 76.3%, and ADE by 28.9%. Compared with the LSTM model, PGRL also improves RMSE, MAE, ADE, and FDE, showing that the residual correction strategy provides additional benefit beyond solely data-driven prediction. In general, the results indicate that the physics-guided residual correction model provides the strongest quantitative performance by combining the structured baseline of the physics model with the adaptive correction capability of the learning-based component.

### Comparative analysis across flight phases

Because UAVs motion is phase dependent, overall trajectory metrics alone are not sufficient to fully evaluate prediction performance. Therefore, the prediction results were also analyzed separately for the three operational phases: takeoff, cruise, and landing. This phase-wise analysis is important because each phase has different motion characteristics and safety implications. Takeoff and landing are short but safety-critical, while cruise usually covers a longer part of the mission and may lead to accumulated prediction drift over time. Table 5 presents the phase-wise performance of the available models. The comparison includes the physics-based model, the LSTM model, MLP fusion, and the proposed PGRL model. The metrics are reported using RMSE, MAE, ADE, and FDE. The evaluation follows the same prediction setup used in the overall analysis, with a 30 s prediction horizon.

**Table 5:** Phase-wise quantitative performance on the respective evaluation sets. Lower values indicate better performance.

Phase	Model	RMSE (m)	MAE (m)	ADE (m)	FDE (m)
Takeoff	Physics-based	**5.559**	4.791	**4.791**	**10.364**
	LSTM	10.663	4.595	13.784	24.274
	MLP Fusion	14.272	12.050	12.050	19.219
	PGRL	7.575	**2.904**	8.710	17.615
Cruise	Physics-based	11.706	7.517	7.517	34.158
	LSTM	2.374	1.027	3.080	3.712
	MLP Fusion	4.153	3.682	3.682	5.038
	PGRL	**1.969**	**0.856**	**2.568**	**3.134**
Landing	Physics-based	6.661	5.343	5.343	11.941
	LSTM	1.823	0.783	2.350	**2.423**
	MLP Fusion	3.700	3.315	3.315	5.298
	PGRL	**1.740**	**0.763**	**2.290**	2.983

The phase-wise results show that the prediction difficulty is not the same across all flight phases. For the learning-based models, takeoff produces the largest errors. The LSTM model gives an RMSE of $10.663~\mathrm{m}$ during takeoff, while the MLP fusion model reports $14.272~\mathrm{m}$ and the PGRL model $7.575~\mathrm{m}$. This indicates that takeoff is the most difficult phase for the learning-based predictors. A likely reason is that takeoff involves rapid vertical motion, acceleration changes, and transition behavior immediately after lift-off. These motion changes make the future trajectory harder to predict. It is also the only phase in which the direct MLP fusion outperforms the residual-correction approach on a subset of metrics; however, the physics-based model attains the best RMSE ($5.559~\mathrm{m}$), the best ADE ($4.791~\mathrm{m}$), and the best FDE ($10.364~\mathrm{m}$) here, while PGRL achieves the best MAE ($2.904~\mathrm{m}$). The weaker takeoff performance is mainly related to the poor initial predictions of the LSTM backbone during this highly dynamic phase. Although the PGRL correction reduces the LSTM error, it cannot fully compensate for the large long-horizon deviation. The limited diversity of takeoff samples, even after phase balancing, also contributes to the remaining error.

For the cruise phase, the physics-based model shows the largest error. This suggests that the standalone physics-based estimation accumulates strong prediction drift during the longer cruise segment, which is mainly caused by the accumulation of model deviation over time. Unlike takeoff and landing, where the motion duration is shorter and more constrained by vertical operation, cruise involves longer horizontal displacement and sustained velocity. Small errors in velocity, heading, drag representation, or wind compensation can therefore accumulate over the prediction horizon and lead to substantial trajectory drift. In contrast, the LSTM and PGRL models perform much better during cruise. The PGRL model achieves the lowest cruise RMSE of $1.969~\mathrm{m}$, MAE of $0.856~\mathrm{m}$, ADE of $2.568~\mathrm{m}$, and FDE of $3.134~\mathrm{m}$, outperforming the LSTM on all four metrics. This shows that the learned correction component is effective in reducing both average long-horizon drift and endpoint error. The landing phase shows the smallest errors overall. The PGRL model achieves the best landing performance on RMSE ($1.740~\mathrm{m}$), MAE ($0.763~\mathrm{m}$), and ADE ($2.290~\mathrm{m}$), while the LSTM attains the best FDE ($2.423~\mathrm{m}$). This indicates that the landing trajectories in the test data are more regular and easier to predict than takeoff. However, landing is still operationally critical, and therefore low prediction error in this phase is important for safe UAV operation near the ground and around landing areas.

In summary, the phase-wise analysis confirms the value of the proposed physics-guided residual-correction framework. The physics-based model alone provides useful motion structure but suffers from large prediction drift, particularly during cruise. The LSTM model substantially reduces this drift by learning temporal motion patterns from data. The MLP fusion model is competitive in the takeoff phase but degrades performance in cruise and especially in landing, where its errors are considerably higher than those of the LSTM. In contrast, the proposed PGRL model provides the most consistent performance across phases: it achieves the best MAE in takeoff, the best values on all metrics in cruise, and the best values on RMSE, MAE, and ADE in landing. These results show that combining physical guidance with learned residual correction improves robustness across different flight regimes and provides more reliable trajectory prediction than the standalone physics-based, LSTM-only, and MLP fusion models.

### Physical feasibility of predicted trajectories

In addition to numerical prediction accuracy, the physical realism of the predicted trajectories was also evaluated. This is important because a model can produce a small position error but still generate unrealistic motion, such as sudden speed changes, high acceleration, large jerk, or unsafe descent behavior during landing. Therefore, four physical-feasibility indicators were used: speed violation rate, acceleration RMS, jerk RMS, and landing descent rate violation. Lower values indicate better physical feasibility.

Table 6 presents the phase-wise physical feasibility results for the evaluated models. The results show a clear difference between prediction accuracy and physical plausibility. The physics-based model gives the most physically consistent trajectories in all phases. It has zero speed violations and the lowest acceleration and jerk values. This is expected because the model is directly based on physical motion assumptions and therefore produces smoother trajectory evolution. The LSTM model improves trajectory prediction accuracy compared with the physics-only model, but its physical feasibility is weaker. It produces non-zero speed violation rates and much larger acceleration and jerk values than the physics-based model. This indicates that the LSTM can learn the observed trajectory patterns from data, but it does not automatically guarantee physically smooth motion. This behavior is visible across all phases, particularly in cruise, where the acceleration and jerk values remain considerably higher than those of the physics-based model. The MLP fusion model shows the weakest physical feasibility among the learned models. It produces the highest speed violation rate in takeoff (0.061) and its acceleration and jerk RMS values are the largest of the learned models in takeoff, where the jerk RMS reaches 383.101. This suggests that direct point-wise fusion can reduce some prediction errors but introduces abrupt point-to-point trajectory changes when physical smoothness is not explicitly constrained. The PGRL model provides the strongest physical-feasibility performance among the learned models. It achieves a zero speed violation rate in every phase, and it gives lower acceleration RMS than both the LSTM and MLP fusion models in all phases. During cruise, PGRL reduces the acceleration RMS to 1.192 and the jerk RMS to 15.812, compared with 11.056 and 54.561 for the LSTM, showing that the physics-informed residual-correction strategy yields a clear smoothness improvement over the purely data-driven model. In landing, PGRL achieves the lowest descent-rate violation among the learned models, with a value of 0.002 compared with 0.032 for the LSTM and 0.030 for the MLP fusion. Overall, its physical-feasibility behavior is the most balanced of the learned models because it combines the lowest speed violation rate, the lowest acceleration RMS, and the lowest descent-rate violation, although a clear gap to the physics-based model remains.

For a compact comparison, Table 7 reports the overall physical feasibility indicators for all models. The results confirm that the physics-based model is the smoothest and most physically realistic model. It has no speed violations, very low acceleration and jerk, and no landing descent rate violation. This confirms the benefit of explicit physical structure for producing stable motion. Among the learned models, the PGRL model provides the best overall physical-feasibility balance. It achieves the lowest speed violation rate, the lowest acceleration RMS, the lowest jerk RMS, and the smallest landing descent violation among the learned models. Compared with the LSTM, PGRL reduces the overall speed violation rate from 0.013 to 0.000, the acceleration RMS from 8.705 to 2.549, and the jerk RMS from 40.483 to 34.734. Compared with the MLP fusion model, PGRL shows a much stronger improvement, reducing the acceleration RMS from 13.470 to 2.549 and the jerk RMS from 178.928 to 34.734. These results indicate that the physics-guided residual correction design improves not only prediction accuracy but also physical feasibility compared with the purely data-driven and simple fusion baselines.

Collectively, the physical feasibility analysis shows that the physics-based model remains the most dynamically smooth because it is explicitly constrained by simplified motion assumptions. However, the proposed PGRL model achieves the best physical realism among the learned models while also maintaining strong prediction accuracy. This supports the main objective of the hybrid framework: to combine the predictive flexibility of learning-based models with improved physical consistency.

**Table 6:** Phase-wise physical-feasibility comparison. Lower values indicate better physical realism.

Phase	Model	Speed violation rate	Acceleration RMS (m/s^2^)	Jerk RMS (m/s^3^)	Landing descent violation
Takeoff	Physics-based	**0.000**	**0.039**	**0.241**	–
	LSTM	0.017	7.419	17.707	–
	MLP Fusion	0.061	25.317	383.101	–
	PGRL	**0.000**	4.441	59.899	–
Cruise	Physics-based	**0.000**	**0.098**	**0.108**	–
	LSTM	0.005	11.056	54.561	–
	MLP Fusion	0.004	9.812	66.715	–
	PGRL	**0.000**	1.192	15.812	–
Landing	Physics-based	**0.000**	**0.068**	**0.648**	**0.000**
	LSTM	0.022	4.596	22.806	0.032
	MLP Fusion	0.020	4.291	21.364	0.030
	PGRL	**0.000**	2.372	33.327	0.002

**Table 7:** Overall physical-feasibility comparison across the four models (N=23,336). Lower values indicate better physical realism. Bold values indicate the best overall result, while underlined values indicate the best result among the learned models.

Model	Speed violation rate	Acceleration RMS (m/s^2^)	Jerk RMS (m/s^3^)	Landing descent violation
Physics-based	**0.000**	**0.079**	**0.392**	**0.000**
LSTM	0.013	8.705	40.483	0.032
MLP Fusion	0.021	13.470	178.928	0.030
PGRL	$\underline{{\mathbf {0.000}}}$	$\underline{2.549}$	$\underline{34.734}$	$\underline{0.002}$

### Comparative analysis of the proposed hybrid model variants

To better understand the source of the performance improvement, an ablation-style comparison was carried out. The purpose of this analysis was to examine whether the final performance gain comes from the physics-based model, the data-driven model, the MLP fusion strategy, or the proposed physics-guided residual correction model. For this reason, the proposed PGRL model was compared with three directly comparable variants: the physics-based model, the LSTM-only model, and the MLP fusion (Tables 4 and 8). The results show that the analytical method is not sufficient to represent the complex UAV trajectory behavior observed in the real flight data. Although the physics-based model provides a structured and physically interpretable motion estimate, its prediction error increases strongly over the prediction horizon. The LSTM-only model substantially improves the prediction accuracy compared with the physics-based model. This confirms that the data-driven model can learn important temporal motion patterns from the telemetry data. However, the LSTM model does not explicitly use the physics-based estimation as a correction baseline and therefore relies mainly on learned trajectory patterns. The MLP fusion model further improves some error metrics compared with the LSTM, particularly RMSE, MAE, and ADE in the overall results. This suggests that learnable fusion of physics-based and data-driven predictions can be effective. However, the MLP fusion model does not consistently improve all metrics, as its FDE is higher than that of the LSTM in the overall comparison. This indicates that direct fusion can improve average trajectory agreement but may not always reduce the final prediction error, especially when the physics-based prediction contains accumulated drift.

**Table 8:** Ablation-style comparison of the proposed hybrid model variants on the test set.

Model variant	Physics prior	Data-driven learning	Residual correction	ADE (m)
Physics-based	Yes	No	No	12.160
LSTM	No	Yes	No	4.707
MLP fusion	Yes	Yes	No	5.584
PGRL	Yes	Yes	Yes	**4.641**

The proposed PGRL model achieves the strongest overall quantitative performance among the evaluated variants. It provides the lowest RMSE, MAE, ADE, and FDE in the overall test set comparison. This shows that the main benefit does not come from physics or data alone, but from using the physics-based prediction as a structured baseline and learning a residual correction from data. In this way, the model can retain useful physical guidance while compensating for the limitations of simplified dynamics. In summary, the ablation comparison confirms the value of the proposed physics-guided residual correction design. The physics-based model provides interpretability and structured motion guidance but suffers from large accumulated drift. The LSTM model captures dominant temporal patterns from data but does not explicitly exploit the physics-based baseline. The MLP fusion model shows that adaptive combination of physics and learning can improve average prediction accuracy, but its performance is not uniformly superior across all metrics. In contrast, the PGRL model provides the most consistent overall improvement by combining physical structure with learned residual correction. This makes the proposed hybrid formulation more effective than the physics-based model, the LSTM model, and the MLP fusion baseline.

### Qualitative trajectory comparison

**Fig. 2:**
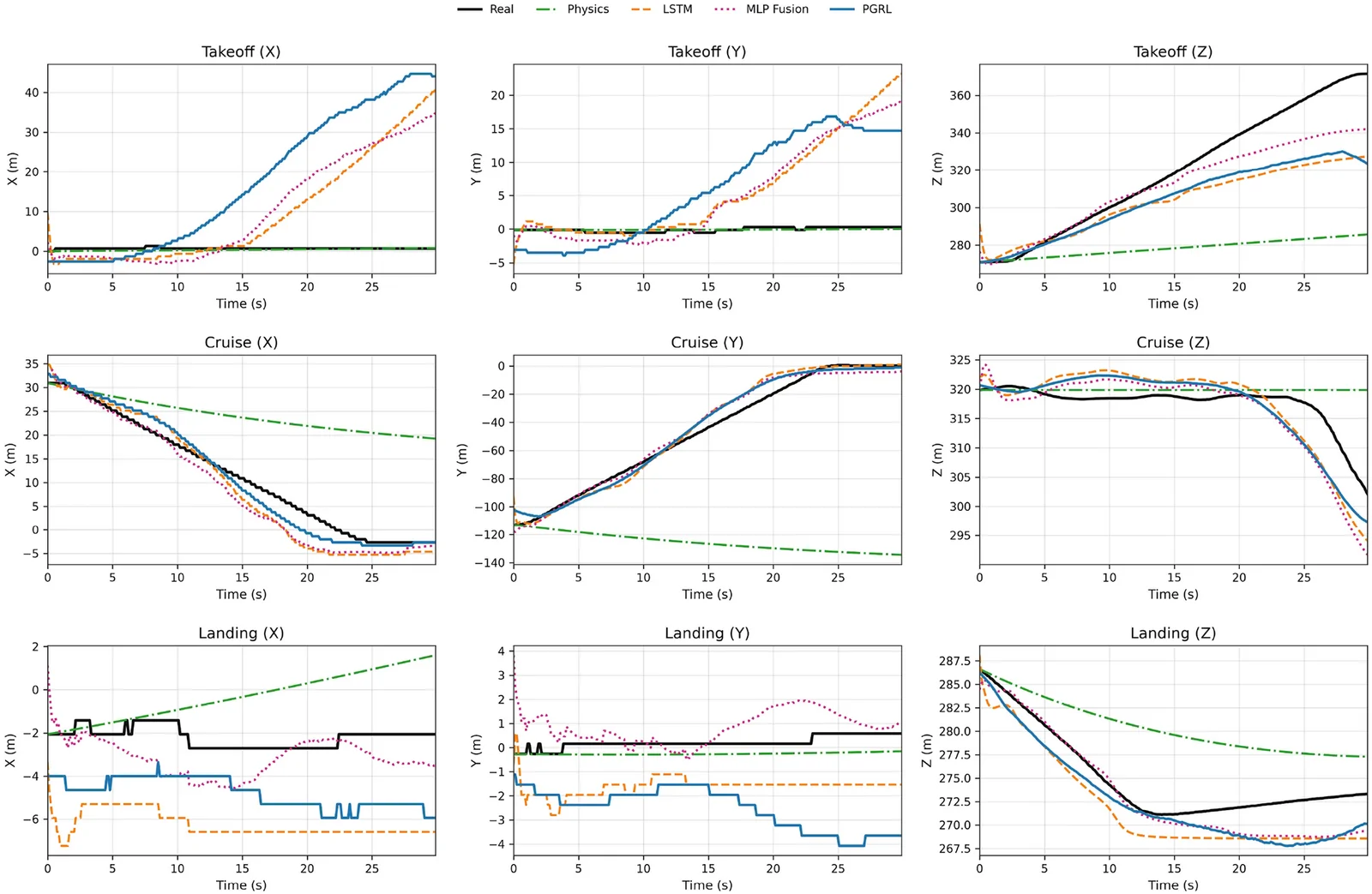
Phase-wise comparison across takeoff, cruise, and landing.

**Fig. 3:**
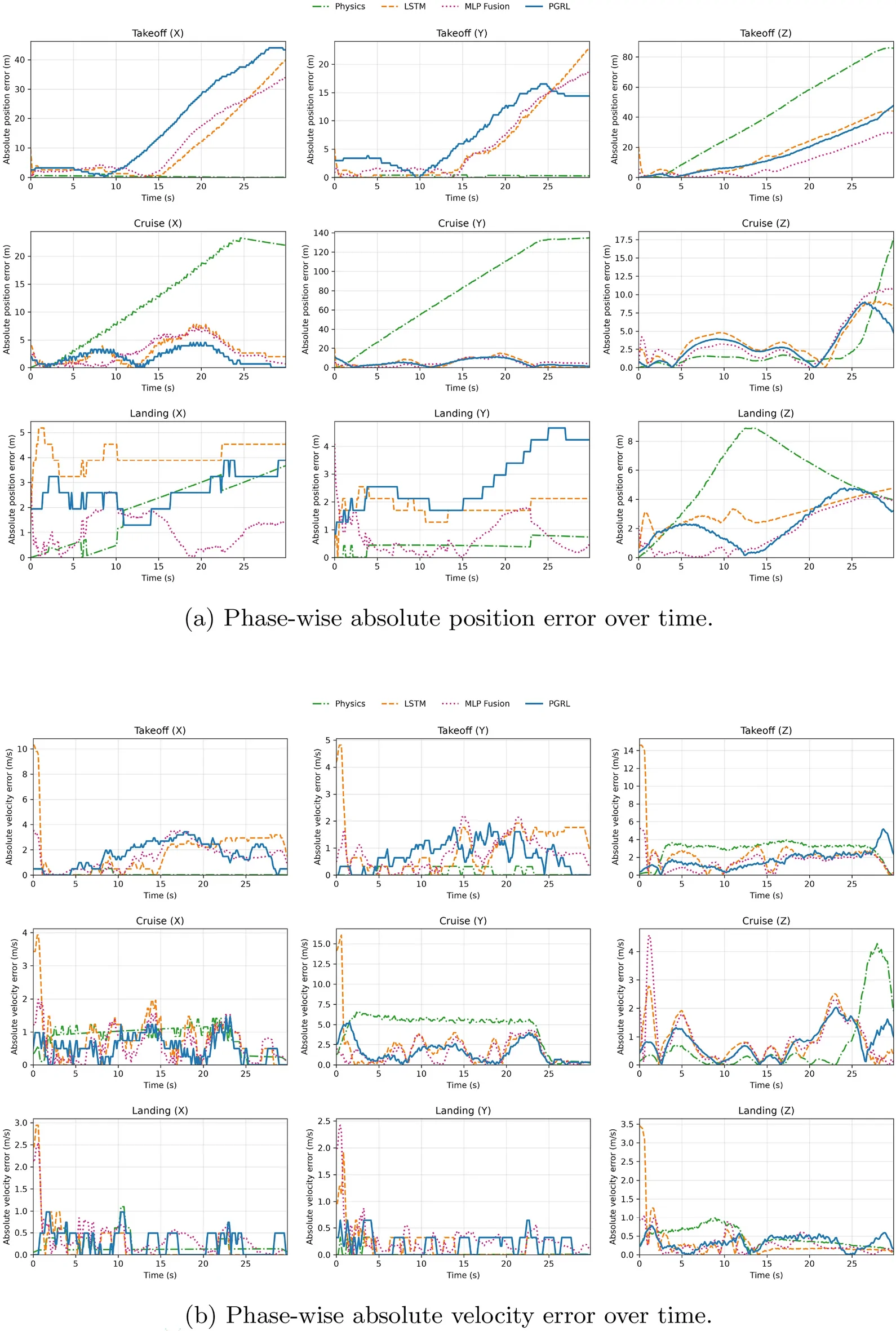
Additional time-series error analysis for the trajectory-prediction models.

**Fig. 4:**
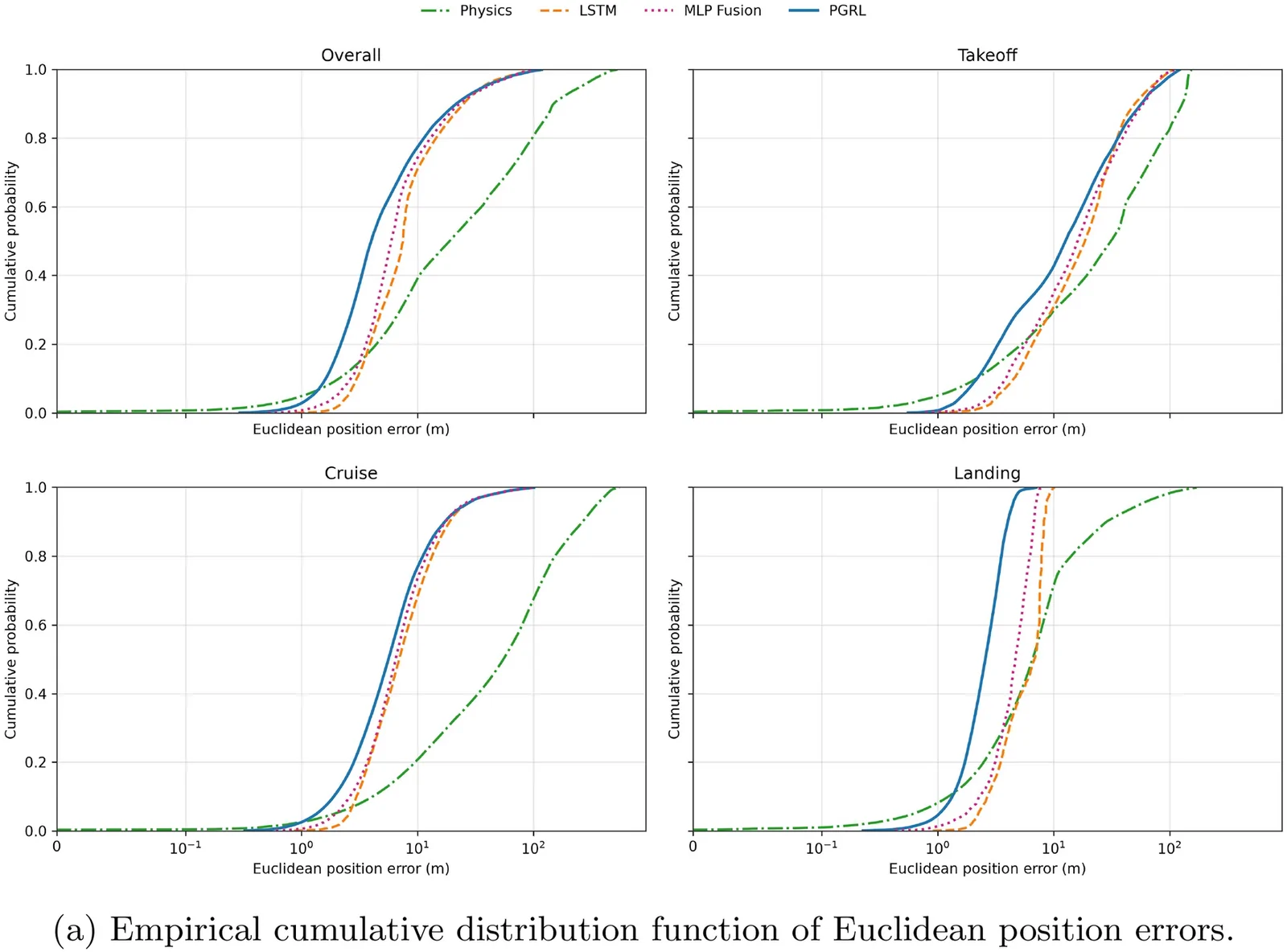
Additional distributional error analysis for the four trajectory-prediction models.

**Fig. 5:**
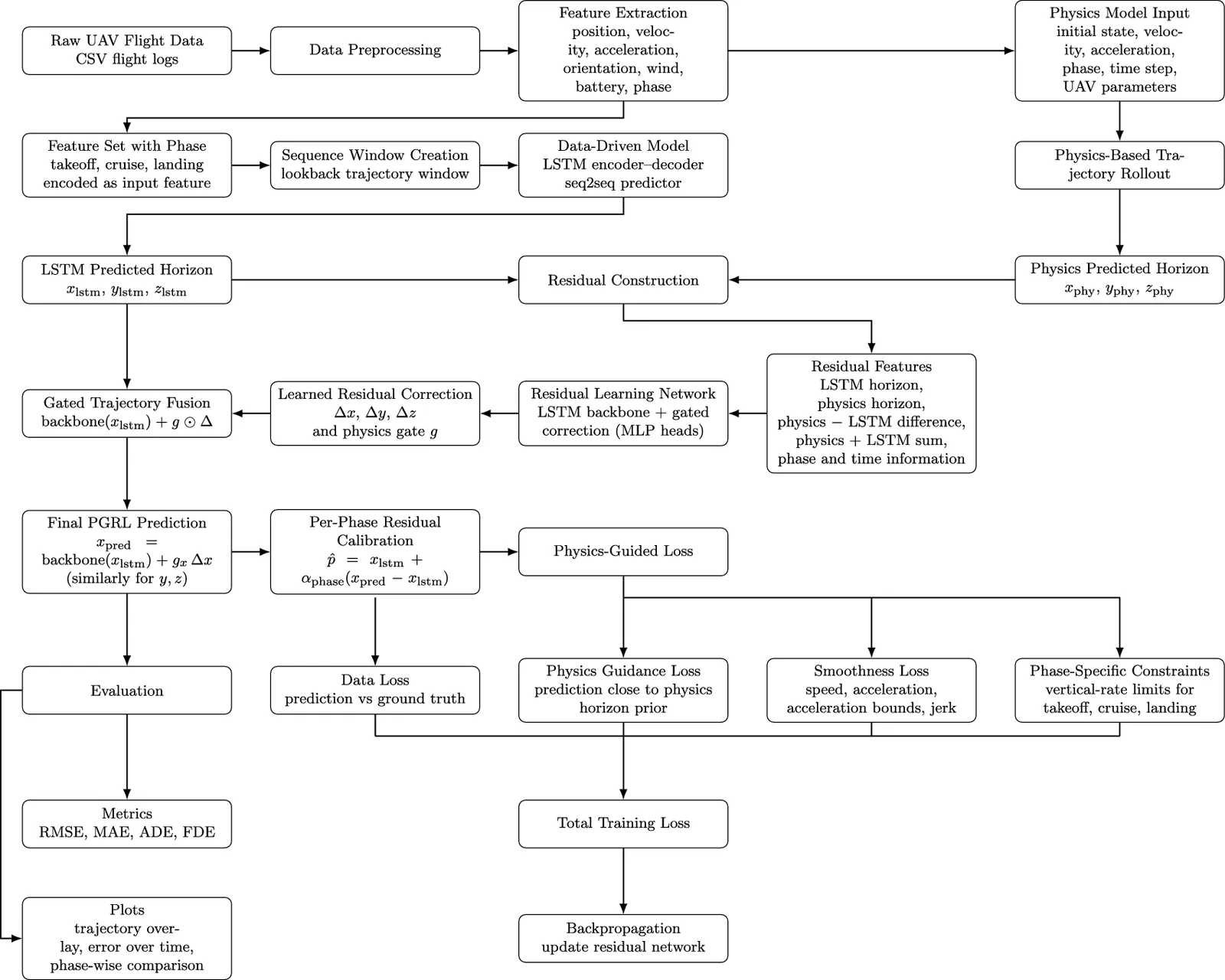
Block diagram of the physics-guided residual learning (PGRL) framework for UAV trajectory prediction.

In addition to numerical evaluation, representative trajectory plots were examined to understand how the predicted paths behave in three-dimensional space. This qualitative analysis is useful because error metrics alone do not show whether a model follows the correct motion direction, captures phase transitions, or produces visually smooth and realistic trajectories (Figs. 2 and 3). The qualitative results support the main findings of the quantitative analysis (Fig. 4). The physics-based model generally produced smooth and physically structured trajectories. However, its predictions often drifted away from the measured trajectory as the prediction horizon increased. This behavior is consistent with the quantitative results, where the physics-based model produced the largest RMSE, ADE, and FDE values. The drift was especially visible in longer trajectory segments, where small modeling errors accumulated over time. The LSTM model followed the measured trajectory more closely than the physics-based model. It reduced the large drift observed in the physics-only prediction and captured the main direction of motion more effectively. However, the LSTM prediction is mainly data-driven, and therefore it can still show local deviations or less regular motion behavior, especially over longer prediction horizons and during phase transitions. The fusion-based prediction reduced some of the limitations of the standalone models. By combining information from the physics-based and data-driven predictions, the fusion approach improved the visual agreement with the real trajectory in several cases, most notably during takeoff, where it achieved the best phase-wise accuracy. However, fusion does not provide the best result in all phases, because the relative importance of the physics model and the learned model changes across takeoff, cruise, and landing; its agreement degrades in cruise and landing. This explains why fusion is less accurate than the proposed residual correction approach in the overall quantitative evaluation.

The proposed PGRL model showed the most consistent qualitative agreement with the measured trajectory among the directly comparable models. Its predicted path remained closer to the real trajectory than the physics-based model and generally improved upon the LSTM prediction. This is consistent with the quantitative results, where PGRL achieved the lowest overall values on all four error metrics. The visual results therefore confirm that the residual-correction strategy helps reduce trajectory drift while preserving a structured prediction form. However, the qualitative results also show that prediction quality can vary across flight phases. Takeoff remains challenging because of rapid vertical motion and acceleration changes. Cruise can lead to accumulated drift in physics-only prediction. Landing requires stable descent behavior near the ground, where even small vertical deviations can be important. Therefore, the qualitative comparison confirms that UAV trajectory prediction should be evaluated not only by overall error values, but also by phase-wise behavior and physical realism.

## Conclusion

### Overview of the research

This paper presented a physics-informed hybrid residual-correction framework for short-horizon UAV trajectory prediction in urban environments. The proposed approach was motivated by the limitations of purely analytical and purely data-driven predictors when applied to realistic UAV operations characterized by wind disturbances, strongly phase-dependent motion, and imbalanced data. To address these challenges, the framework combined a physics-based trajectory model with a data-driven sequence model and used a residual-correction strategy to learn the mismatch between analytical prediction and observed trajectory behavior. In addition, the model was trained with physics-informed feasibility regularization to encourage realistic speed, acceleration, jerk, and landing descent behavior. The results lead to several important conclusions about physics-based, data-driven, and hybrid UAV trajectory prediction. First, neither the physics-based model nor the data-driven model alone is sufficient to achieve the best overall performance. The physics-based model produced the smoothest and most physically plausible trajectories, but its position accuracy was limited. In contrast, the LSTM model improved prediction accuracy, but its trajectories were less physically regular and showed more speed, acceleration, and jerk violations. This shows that the two approaches provide complementary strengths. Second, the results confirm that hybrid prediction is more effective than standalone prediction, provided the integration is properly designed. The proposed PGRL model outperformed the physics-only and LSTM-only models across all overall accuracy metrics, supporting the main idea of the study: physics-based prior information and data-driven learning should be combined rather than treated as separate alternatives. Third, not all hybrid designs behave in the same way. The direct MLP fusion degraded overall RMSE, MAE, ADE, and FDE relative to the LSTM and produced the least physically feasible trajectories, with high speed-violation rates and large acceleration and jerk values; even in the takeoff phase, where it was most competitive, it did not achieve the best accuracy on every metric. In contrast, the structured multi-step PGRL formulation achieved the best overall accuracy on all metrics, the best phase-wise performance in cruise and landing, and the strongest physical feasibility among the learned models. This indicates that how physics and learning are combined matters as much as whether they are combined. The phase-wise analysis further showed that prediction difficulty depends strongly on the flight phase. Cruise and landing were handled well by the hybrid models, while takeoff remained the most difficult phase for all learning-based predictors. In addition, the physical-feasibility analysis showed that lower numerical error does not automatically mean more realistic motion.

Finally, the findings suggest that future work should focus on improving physical consistency and phase robustness, particularly during takeoff, while preserving the strong predictive accuracy. This could be addressed through a hybrid framework with enhanced phase-aware modeling, improved residual design, stronger ascent constraints, and unified training that jointly optimizes prediction accuracy and dynamic realism.

### Limitations of this study and future work

Despite the encouraging results, several limitations should be acknowledged. First, the physics-based model is considered simplified to remain computationally efficient. While practical, this means that some important effects, such as full controller dynamics, actuator lag, detailed aerodynamics, and complex urban wind interaction, are not explicitly modeled. As a result, part of the prediction error may come from limitations in the physics prior itself. Second, the current study focuses mainly on deterministic short-horizon position prediction. Although physical feasibility is considered in the training loss, the results do not yet include a full and complex set of explicit feasibility statistics such as speed-violation rate, jerk distribution, or landing descent-rate compliance. Future work should vary the penalty coefficients individually and jointly to examine their effects on RMSE, ADE, FDE, speed violations, acceleration, and jerk, and to identify configurations that provide an appropriate accuracy feasibility trade-off for different UAV operations. Third, this study does not examine each part of the PGRL model separately during takeoff, so the exact cause of the remaining error cannot be confirmed. Future work should analyze the gate values, correction size, and backbone output to identify where the problem occurs. It should also test stronger takeoff-specific loss weighting, better correction models, and more varied takeoff data. Fourth, the evaluation is limited to the available dataset and model settings. The generalization of the proposed framework to other UAV platforms, payload conditions, wind environments, or urban settings has not yet been fully tested. Another limitation of this study is that wind disturbances are modeled using the available wind-speed and wind-direction measurements as a constant wind vector over the forecast horizon. The model does not explicitly predict the stochastic time history of gusts or turbulence. Therefore, the residual-learning component should be interpreted as learning correction patterns under the wind conditions represented in the dataset rather than as a general model of unseen wind disturbances. Future work should evaluate the framework under controlled wind regimes, stronger gusts, and different turbulence spectra. Another limitation of the present study is that the experimental evaluation is based on a single dataset. The proposed PGRL methodology is intended as a general hybrid modeling framework; however, its physics-based component contains platform dependent parameters, including vehicle mass, inertia, thrust limits, aerodynamic coefficients, reference areas, and phase-specific operating bounds. Consequently, evaluation on a different UAV platform would require re-parameterization or system identification of the corresponding physics model, followed by retraining or adaptation of the learned components. Future work could evaluate the framework using independently collected datasets, platform-specific physical parameters, and cross-environment or cross-platform training and testing protocols.

Several directions remain for future work. Future work could extend the proposed UAV trajectory prediction framework toward a digital twin (DT) enabled real-time deployment architecture. Although the present study evaluates the LSTM, physics-based, MLP-fusion, and PGRL models using offline flight-log data, the developed prediction models can serve as the predictive intelligence layer of a future DT environment. In this framework, online UAV telemetry, including position, velocity, acceleration, attitude, angular velocity, wind conditions, battery state, and flight phase, would be continuously synchronized with a virtual model. The DT environment would represent the UAV state, surrounding buildings, vertiport layout, obstacle geometry, wind fields, and safety zones. At each update cycle, the latest telemetry window would be used to generate a short-term trajectory forecast, for example, over a 30-second prediction horizon. The physics-based model would provide a physically consistent rollout, while the LSTM would capture learned motion patterns from historical data. The MLP fusion or PGRL residual-correction module would then combine these outputs to generate a robust future trajectory prediction. The predicted trajectory could be evaluated inside the DT environment using uncertainty propagation, obstacle-proximity analysis, and collision probability estimation. This would enable online safety monitoring, safe corridor identification, risk assessment, and vertiport operation support.

A further research direction concerns the geometric structure and input sensitivity of the learned residual correction. The present PGRL model generates a deterministic horizon-wise correction from the joint physics-based and data-driven predictions, but it does not explicitly constrain the internal correction representation to follow a particular geometric structure. Geometry-aware parameterizations, such as separating the residual into direction and magnitude components, could potentially improve interpretability and phase-wise transfer. Future work could therefore investigate spectral normalization, Jacobian-norm penalties, or perturbation-consistency regularization to reduce sensitivity to small input variations and improve correction stability, particularly during the highly dynamic takeoff phase.

Future deployment should also investigate real-time telemetry, latency, model update frequency, online model adaptation, and closed-loop validation. Therefore, the proposed framework provides a foundation for transitioning from offline UAV trajectory prediction toward DT-enabled real-time risk-aware operation in urban air mobility environments.

## Figures and Tables

**Algorithm 1:**
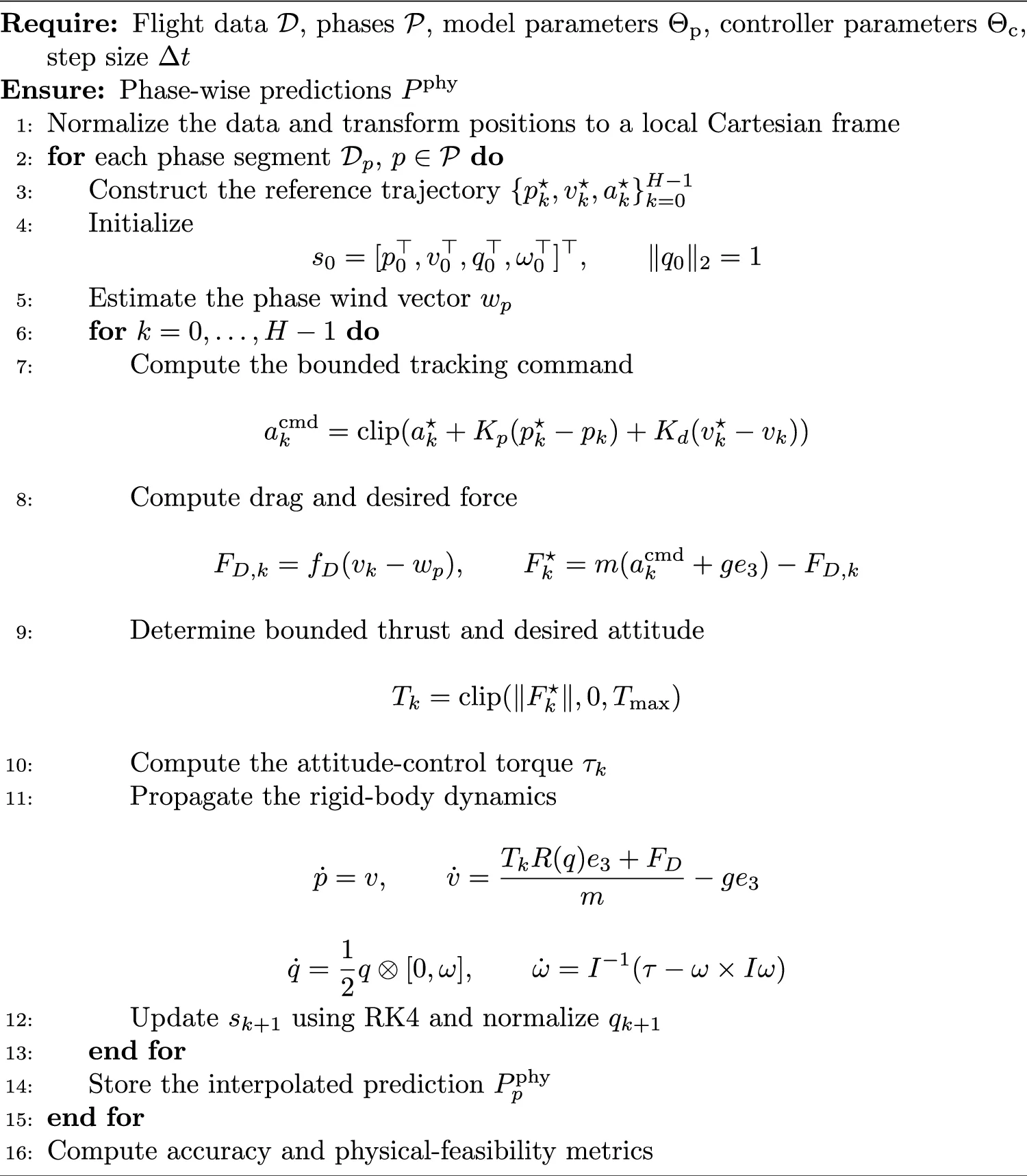
Physics-Based Prediction

**Algorithm 2:**
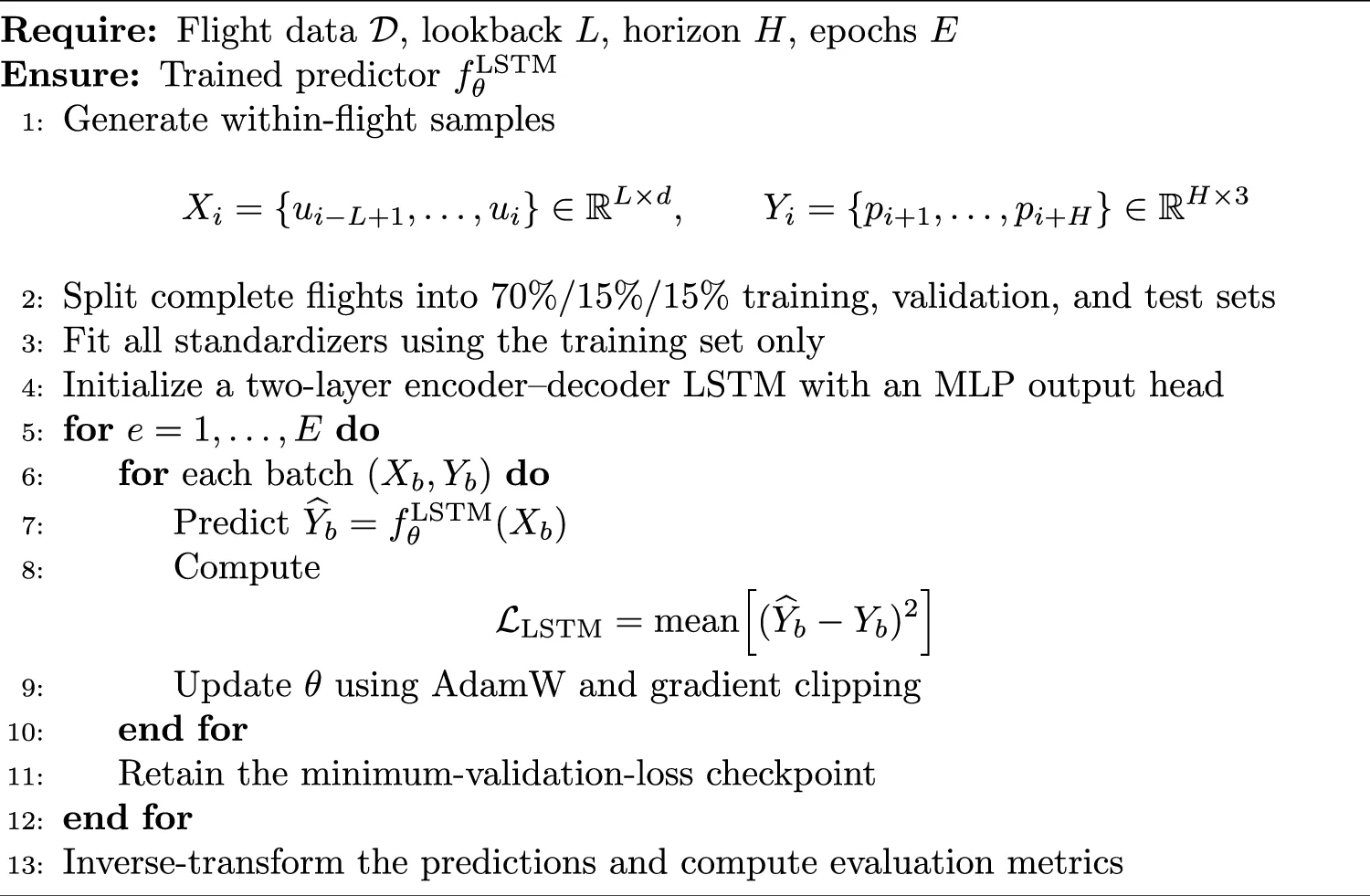
Multi-Step LSTM

**Algorithm 3:**
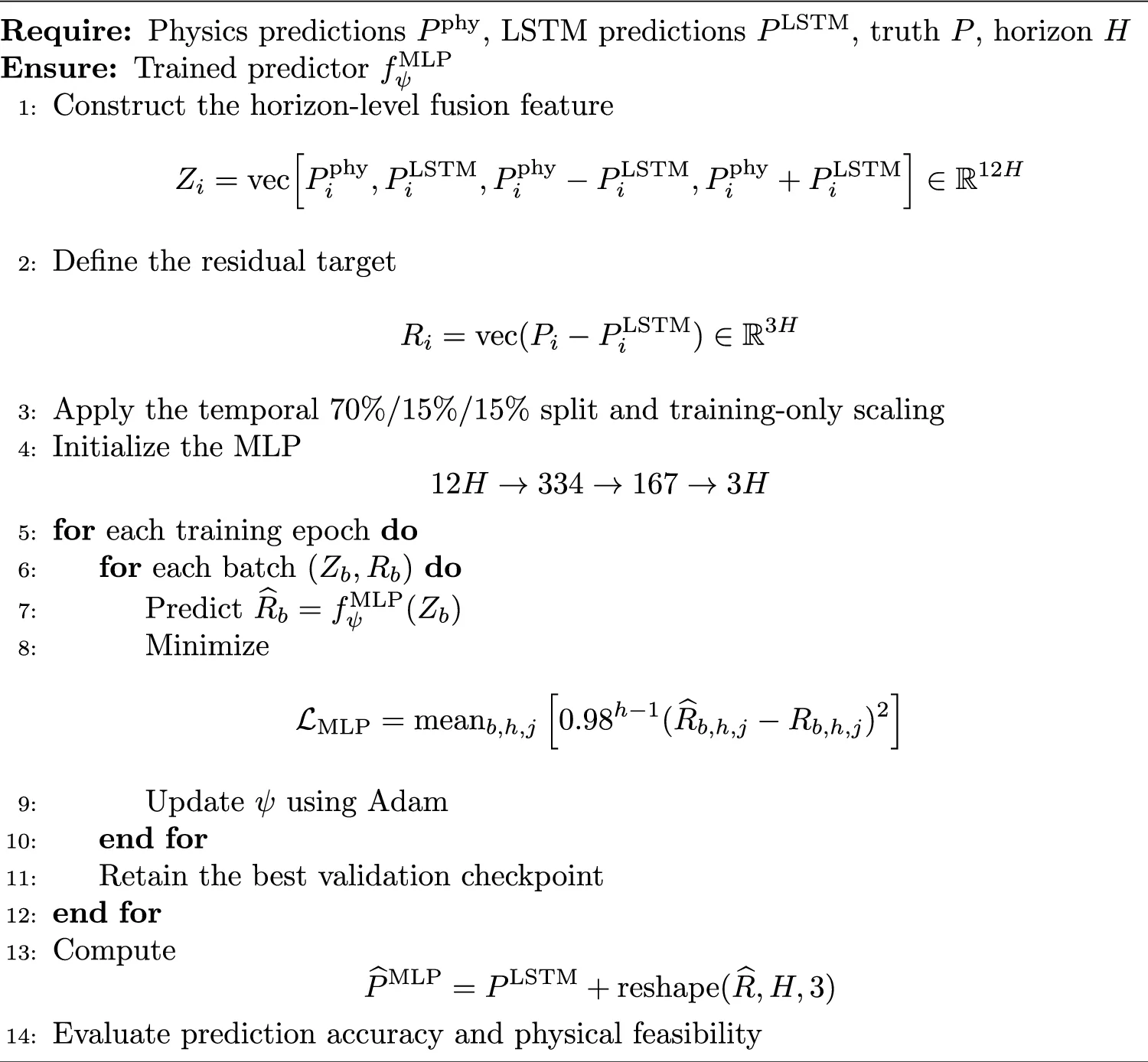
MLP Fusion

**Algorithm 4:**
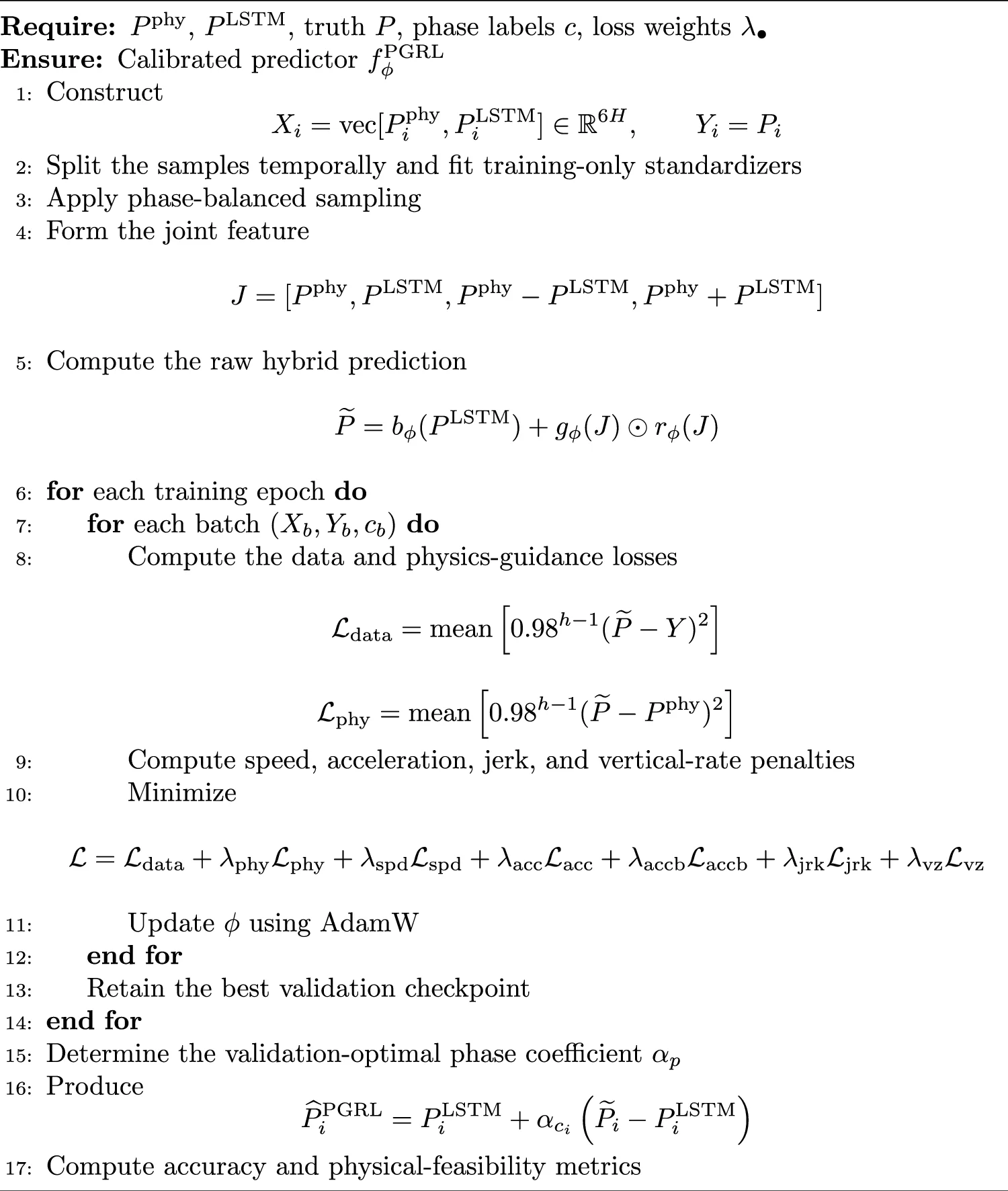
Physics-Guided Residual Learning

**Fig. 6:**
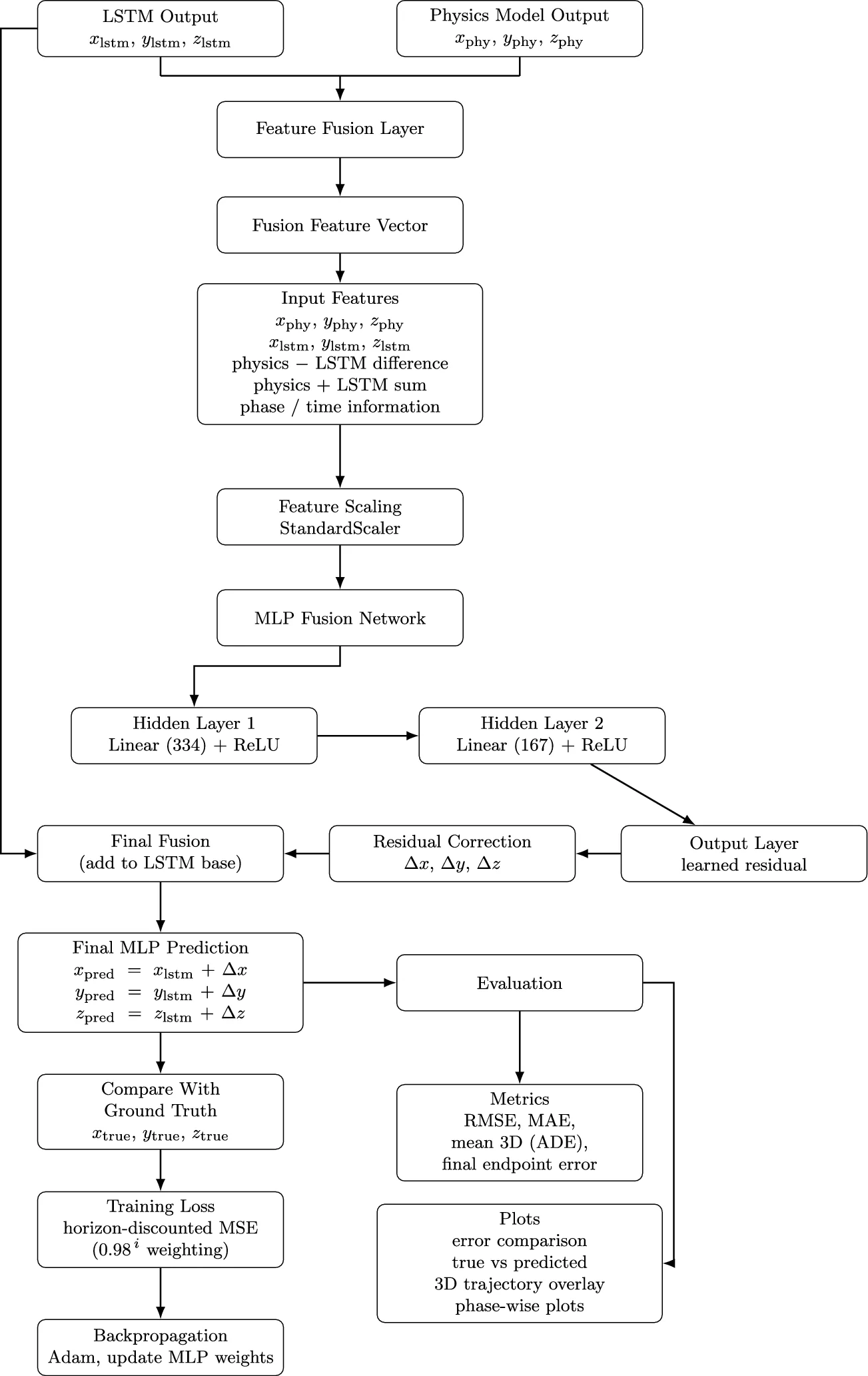
Block diagram of the MLP-based trajectory-fusion framework.

**Fig. 7:**
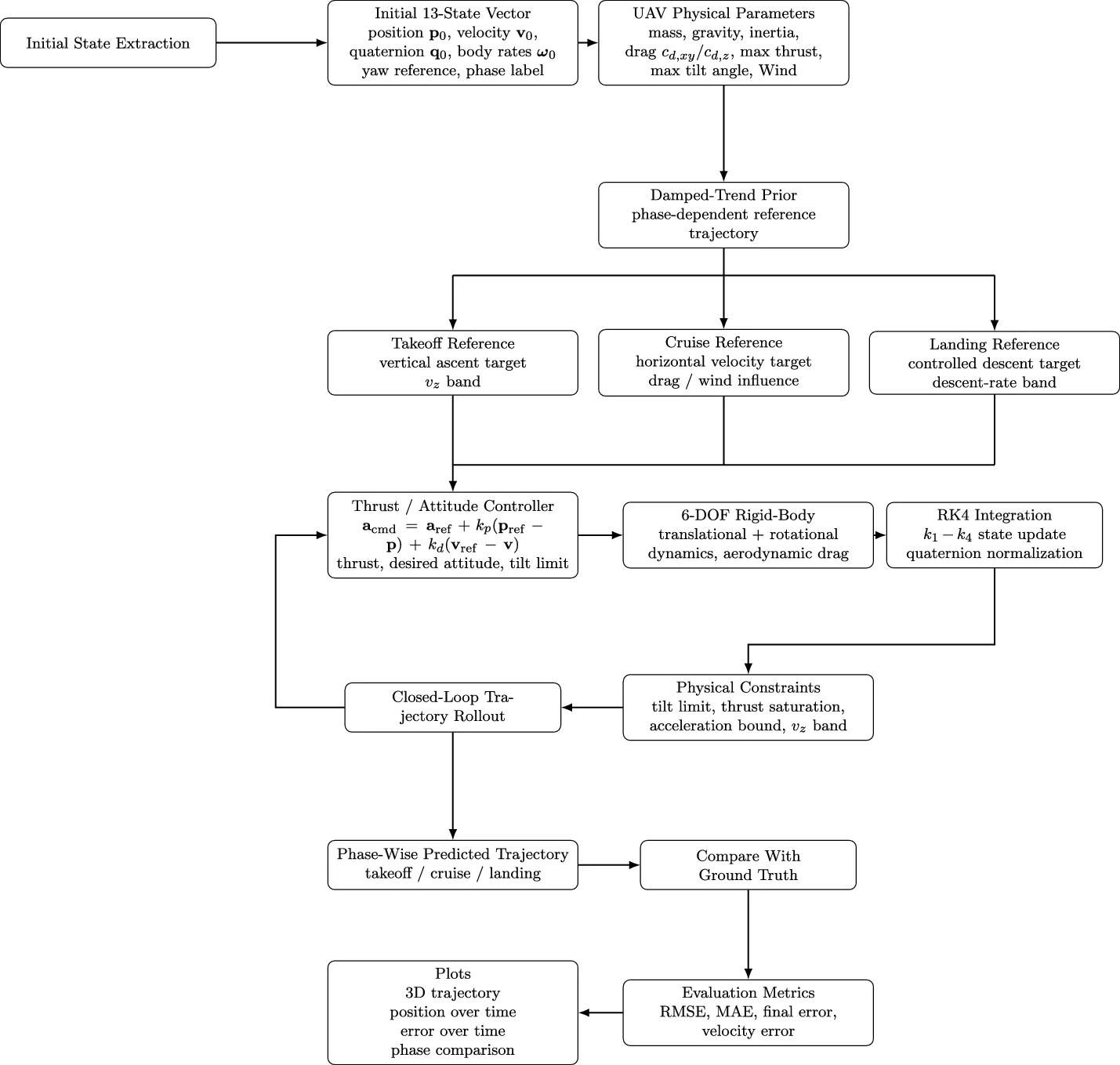
Block diagram of the phase-aware physics-based UAV trajectory prediction framework.

**Fig. 8:**
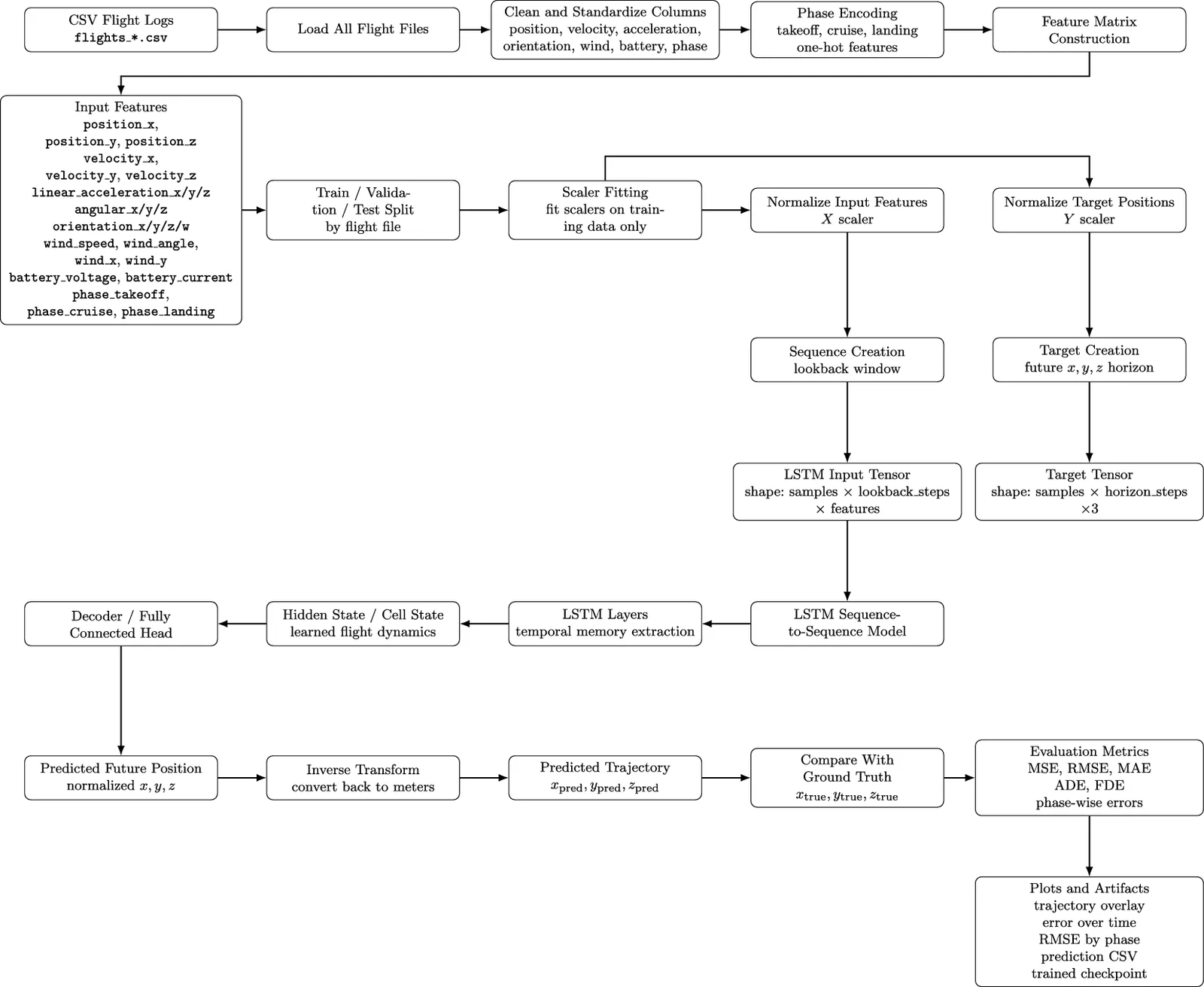
Block diagram of the LSTM sequence-to-sequence framework for UAV trajectory prediction.

## Data Availability

The data is openly available.

## Appendix A: Sensitivity analysis of the prediction models

See Table 9.

**Table 9:** Sensitivity of PGRL performance to physics priors.

Assumed prior	RMSE (m)	MAE (m)	ADE (m)	FDE (m)	RMSE change
Nominal *m=3.6* kg	4.323000	1.395000	4.185000	6.370000	*0.000000%*
Mass 2 kg	4.323011	1.395002	4.185009	6.370128	*+0.000254%*
Mass 6 kg	4.322993	1.394998	4.184995	6.369919	*-0.000162%*
Drag *0.75× *	4.322991	1.394997	4.184993	6.369890	*-0.000208%*
Drag *1.25× *	4.323010	1.395002	4.185008	6.370112	*+0.000231%*
Wind gain 0.10	4.322969	1.394994	4.184984	6.369895	*-0.000717%*
Wind gain 0.50	4.323113	1.395021	4.185067	6.370425	*+0.002614%*
Warm-up 8 s	4.311669	1.387972	4.163800	6.439030	*-0.263399%*
Warm-up 12 s	4.350586	1.402551	4.207534	6.361421	*+0.636823%*
Trend decay $\tau _a=32$ s	4.320155	1.394271	4.182695	6.370843	*-0.067107%*
Trend decay $\tau _a=48$ s	4.325151	1.395705	4.186997	6.370235	*+0.048477%*
takeoff scale $\gamma _{to}=0.24$	4.305686	1.389720	4.169042	6.372680	*-0.401793%*
takeoff scale $\gamma _{to}=0.36$	4.345103	1.400997	4.202872	6.368928	*+0.509987%*
Cruise scale $\gamma _{cr}=0.08$	4.323058	1.395109	4.185209	6.370539	*+0.000044%*
Cruise scale $\gamma _{cr}=0.12$	4.323053	1.395110	4.185213	6.370418	*-0.000066%*
Landing scale $\gamma _{la}=0.16$	4.323056	1.395109	4.185209	6.370478	*0.000000%*
Landing scale $\gamma _{la}=0.24$	4.323056	1.395110	4.185211	6.370478	*+0.000011%*
Acceleration clip 0.32 m/s^2^	4.313035	1.392096	4.176172	6.371589	*-0.231786%*
Acceleration clip 0.48 m/s^2^	4.323056	1.395110	4.185210	6.370478	*0.000000%*

## Appendix B: Additional results of the models

See Figs. 3 and 4.

## Appendix D: Block diagram of the models

See Figs. 5, 6, 7, 8.
